# Structures, Properties, and Performances—Relationships of Polymeric Membranes for Pervaporative Desalination

**DOI:** 10.3390/membranes9050058

**Published:** 2019-05-01

**Authors:** Nayan Ranjan Singha, Mrinmoy Karmakar, Pijush Kanti Chattopadhyay, Sagar Roy, Mousumi Deb, Himarati Mondal, Manas Mahapatra, Arnab Dutta, Madhushree Mitra, Joy Sankar Deb Roy

**Affiliations:** 1 Advanced Polymer Laboratory, Department of Polymer Science and Technology, Government College of Engineering and Leather Technology (Post Graduate), Maulana Abul Kalam Azad University of Technology, Salt Lake City, Kolkata 700106, West Bengal, India; mrinmoy.enterprise@gmail.com (M.K.); mousumidev1994@gmail.com (M.D.); himaratimondal236@gmail.com (H.M.); mmahapatra101@gmail.com (M.M.); duttaarnab1991@gmail.com (A.D.); j.s.d.roy@gmail.com (J.S.D.R.); 2 Department of Leather Technology, Government College of Engineering and Leather Technology (Post Graduate), Maulana Abul Kalam Azad University of Technology, Salt Lake City, Kolkata 700106, West Bengal, India; madhushreemitra84@gmail.com; 3 Department of Chemistry & Environmental Science, New Jersey Institute of Technology, Newark, NJ 07102, USA

**Keywords:** fabrication and properties of inorganic membranes, nanocomposite membrane, physicochemical alterations of desalination membranes, hollow-fiber supported composite membrane, pervaporative desalination-mechanism

## Abstract

For the fulfilment of increasing global demand and associated challenges related to the supply of clean-and-safe water, PV has been considered as one of the most attractive and promising areas in desalinating salty-water of varied salinities. In pervaporative desalination, the sustainability, endurance, and structural features of membrane, along with operating parameters, play the dominant roles and impart paramount impact in governing the overall PV efficiency. Indeed, polymeric- and organic-membranes suffer from several drawbacks, including inferior structural stability and durability, whereas the fabrication of purely inorganic membranes is complicated and costly. Therefore, recent development on the high-performance and cost-friendly PV membrane is mostly concentrated on synthesizing composite- and NCP-membranes possessing the advantages of both organic- and inorganic-membranes. This review reflects the insights into the physicochemical properties and fabrication approaches of different classes of PV membranes, especially composite- and NCP-membranes. The mass transport mechanisms interrelated to the specialized structural features have been discussed. Additionally, the performance potential and application prospects of these membranes in a wide spectrum of desalination and wastewater treatment have been elaborated. Finally, the challenges and future perspectives have been identified in developing and scaling up different high-performance membranes suitable for broader commercial applications.

## 1. Introduction

The world-wide demand of pure water is continuously increasing because of the exponential increase in population, urbanization, and industrialization. Such enhanced water demand is deteriorating the availability of pure water that should impart the greatest threat in the recent future. Instead of the coverage of more than 70% of Earth’s surface by water, only 3% is available as fresh water, of which a very small fraction is accessible to human beings, since the major portion of such freshwater is stored as frozen glaciers and is accumulated in deep underground water reservoirs. As per the recent survey of USA, 2–7 billion people will face water scarcity by the middle of the 21^st^ century, as surface water is often contaminated by various hazardous contaminants, such as dyes, heavy metal ions, salts, and other organic/inorganic contaminants [1,2,3,4,5,6,7,8]. To mitigate such an alarming issue, membrane-based desalination and waste-water recycling can be thought to be one of the most effective technologies to offer safe and cleaner water [9,10]. Membrane, a permselective barrier, allows the selective passage of a particular component from the mixture(s). Membrane-based technologies are one of the fastest growing separation technologies, extensively utilized in desalination, environmental remediation, green energy, food, and chemical and pharmaceutical sectors [11,12,13,14,15,16]. In this context, high TDS concentration beyond 50,000 ppm of saline-, brackish-, and sea-water restricts the uses of various conventional desalinating membrane processes, such as pressure-driven (i.e., RO, NF, UF, and MF), osmotically-driven (i.e., FO and PRO), because of the excessively high hydraulic pressure required to overcome the osmotic pressure of the feed solution [17,18,19,20,21,22]. In order to overcome such problem, the development of non-pressure driven processes, such as MD [23,24,25] and most recently PV [26,27,28,29,30,31,32,33,34,35,36,37,38,39,40,41,42,43], have been developed.

In MD, a hydrophobic microporous membrane is necessary to act as the barrier against the entrance of feed liquid through membrane pores. At the same time, the membrane pores allow water vapor to pass through. However, membrane fouling and wetting are the major challenges encountered in MD processes. As a result of prolonged usage, hydrophobicity of microporous membrane used in MD is deteriorated, resulting in the increment of membrane wetting. Moreover, the pores of hydrophobic membrane can easily be clogged via colloidal and particulate deposition on the membrane surface, thereby affecting the salt rejection ability [44,45]. In addition, membrane fouling may occur via accumulation and blockage of membrane pores by biological matters susceptible to putrefaction or rotting. 

**Figure 1:**
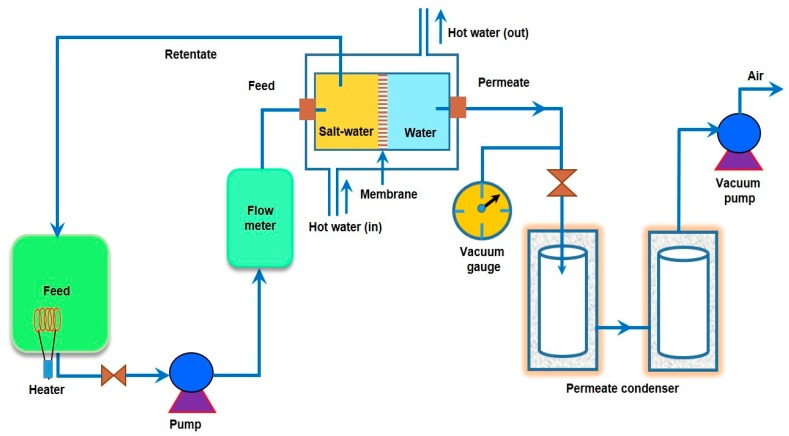
PV desalination apparatus.

Unlike MD, a hydrophilic dense membrane or molecular sieving membrane is utilized as basic component during PV desalination. Accordingly, the hydrophilic membrane preferentially allows water molecules to permeate, discouraging superficial deposition of relatively hydrophobic components on the membrane, leading to the reduced possibility of membrane fouling. Moreover, any hydrophilic membrane does not allow hydrophobic volatile VOCs to permeate through. Therefore, PV is an attractive alternative for extensive removal of even small quantity of VOCs from the feed water. Thus, water vapor can only be permeated through the hydrophilic membrane, followed by condensation of the permeated water vapor in the collection chamber (Figure 1). During PV, passage through the membrane is actuated by the vapor pressure difference between feed and permeate sides. Such difference in vapor pressure is generally created by maintaining either vacuum or by sweeping an inert gas on the permeate side of the membrane [46].

Additionally, PV suffers from some limitations including lower flux as compared to MD. Therefore, for improving permeation flux, modification of PV membranes is essential to provide good affinity and suitable molecular structure for water transport [41,42]. Additionally, there is no need to overcome the osmotic pressure of the feed solution and create a suitably high osmotic gradient across the membrane for attaining a reasonable flux. Therefore, PV works brilliantly for desalinating high-salinity brines and is more foul-resistant compared to the conventional pressure-driven desalination techniques [47].

Desalination of seawater was previously carried out using MSFD and RO. Due to the high energy-demand of MSFD, RO is becoming more useful for seawater desalination [48]. Approximately, 60% of the total desalination plants of the world are RO-based [49]. According to Veolia Environment, the RO market share is expected to climb up to 70% of the total desalination by 2020. In RO, the passage of pure water is allowed to come out from solution using higher mechanical pressure on the feed side as compared to the osmotic pressure of feed solution. However, in RO, pre-treatment and pressurization of raw seawater up to 5000 kPa is essential. Indeed, because of the attainment of biofouling, life-time of polymeric membranes is reduced. Additionally, the membranes have poor recyclability and low chemical stability. Moreover, the typical desalinating efficiency of RO system is only within 35–50% [50]. Furthermore, for RO-based desalination of concentrated sea water, formation of a byproduct causes secondary pollution [51]. Therefore, there is a serious demand for the development of new, cost-friendly, and recyclable membrane-based seawater desalination technology to replace RO [52]. In this regard, membrane-based pervaporative desalination techniques involving thermomechanically and chemically sustainable membranes showing high salt-rejection efficiencies are gaining high insight and paramount impact. Desalination is highly efficient for selective removal of unwanted salt species from non-potable saline sources, such as brackish- (i.e., 1–10 g L^−1^), sea- (i.e., 35 g L^−1^), and brine- (i.e., 75–150 g L^−1^) water [53,54,55,56].

PV is a membrane-based process, which involves the selective separation/rejection of liquids/solids from their mixtures through a dense asymmetric membrane. Except desalination, PV has been extensively used for the systems, which are difficult to be separated through the existing separation processes, such as distillation, adsorption, and extraction. In fact, the use of PV for separating azeotropic and close boiling liquids, heat sensitive materials, and organic mixtures, along with the removals of dilute VOCs from wastewater and recovery of volatile aroma compounds from fruit juices, can be found in literature [57,58,59,60,61,62,63,64]. Rubber membrane-based separation of hydrocarbon from its alcohol mixture was firstly studied by Kahlenberg in early 1906 [65]. Thereafter, frequent utilization of PV has been monitored because of its eco-/cost-friendly performance potential and simple design. For pervaporative desalination, the membranes should possess high hydrophilicity, facilitating the selective permeation of water molecules through the membrane by the solution-diffusion mechanism, resulting in the salt rejection. Mass transport is governed by ΔVP between feed and permeate streams, achieved by maintaining a low continuous pressure on the permeate side of the membrane [66]. Such controlled pressure on the permeate side is maintained by a dry and cold sweeping gas or simply by applying vacuum. The primary problems associated with the pervaporative desalination of seawater are the requirements of pre-treating and heating and the condensation of permeate. Recently, the use of solar thermal collectors reduced the problem of feed-heating [67]. Undoubtedly, PV is an auspicious contender for seawater desalination. Significantly, the use of pervaporative desalination results in the formation of ultra-high purity water in the laboratory and seawater desalination at the small-scale.

Due to the complex mechanism, few empirical models, such as the pore flow model, total solvent volume fraction model, and solution-diffusion model [68,69,70,71,72,73], have been employed to explain the PV mechanism. Of these, solution-diffusion model, originally proposed by Rautenbach et al., resembles the most real PV phenomenon. The solution-diffusion model considers the fugacity gradient between the two sides of the membrane [74]. Indeed, solution-diffusion model was modified further by Mizsey et al. and used in pervaporative dehydration of alcohols [75,76]. Such a model exhibited fair applicability for low feed water concentrations [77]. Since, industrial PV systems are operated in both high and low concentration ranges, the design of more accurate model is essential. Valentínyi et al. modified further the solution-diffusion model to work within high and low concentration ranges [78].

PV-based desalination occurs in three stages: firstly, the adsorption of water into the membrane, followed by the diffusion of the absorbed water through the membrane evaporation into vapor phase on the other side of membrane and, finally, condensation of the water vapor to produce fresh water. The PV-based desalination is advantageous because of high salt rejection and simple instrumentation of high-salinity feed solutions. In fact, salt rejection efficiency of PV-based desalination for monovalent salts is ~99% and is independent of the operating conditions because of the non-volatility of salt, high selectivity of membranes, and high density of hydrophilic polymeric membranes or the tunable pore sizes of inorganic membranes [79]. Chemical properties of the membrane materials play the pivotal role in designing high-performance membranes for pervaporative desalination. The most commonly used PV membranes are composed of different types of materials, such as polymer (i.e., crosslinked PVA), inorganic (i.e., NaA zeolite), and polymer-inorganic hybrid materials (i.e., PVA-MA) [80,81,82,83,84,85]. 

Recent developments in separation and purification technologies coupled with advanced nanotechnology are becoming one of the most potential areas for resolving burning issues of water decontamination. The emerging tailor-made nanostructured membranes are continuously changing the concept of separation techniques, building up new methodologies, which surpass the conventional achievements. Though novel nano-enabled composite membranes have attracted wide attention in UF [86,87,88], NF [89,90], and FO [91], few studies have addressed their potential applications towards PV-based desalination. The quest for the development of superior membrane materials to overcome the ‘trade-off’ relationship in membrane separation is going on. Fabrication of novel polymeric materials and distinct nanomaterials, and the development of cost-effective superior stable modular design open the path towards the feasible commercialization of PV separation system. This review article discusses the current research and development of polymeric membranes for desalination via PV in relation to their synthesis and fabrication techniques, application approaches, challenges, and possibilities.

## 2. A Brief History of Membranes in Pervaporative Desalination

Desalination had been used thousands years ago by the Greek sailors to obtain fresh water via boiling of saline water and the Romans to trap salt using clay filters. The desalination of sea water to obtain drinking has a long and rich history (Table 1). Desalination was firstly used by Aristotle in 350 B.C.E. Man has always considered the sea for the hope of obtaining drinking water. The concept of desalination is based on evaporation and was initially proposed centuries ago. However, it could not be incorporated into boats until the sixteenth century, allowing them to be self-sufficient in the event of an emergency. Before World War-II, desalination-based evaporation was frequently employed in boats, crossing oceans on long trans-Atlantic voyages. In this context, MSFD, the first large-scale modern desalinating process, was evolved in 1955 in USA. Although MED had been discovered earlier and had greater potential than MSF, it took longer for the MED process to be efficient industrially. The first MED plant was constructed in Aruba in 1959. The modern sophisticated methods still use the concepts of distillation (Figure 1). There are about 15,000 desalination plants around the world, of which the biggest plants are mostly located in UAE, Saudi Arabia, and Israel. Australia’s first major seawater desalination plant was commissioned in Perth in 2006 that has the capacity of producing 45 billion liters per year.

Distillation-based technologies remained the primary approach to water desalination until the development of membranes, though such techniques suffer from certain limitations (Table 1). One such major limitation includes thermal desalination, which consumes a very high amount of energy and is, therefore, replaced by RO membranes. Membrane technologies arose because of a breakthrough in the use of polymer films for separating salt from water in the late 1950s/early 1960s. Reid and Breton in 1959 [92] first demonstrated the possibility of desalination using polymeric cellulose films, which led to the development of the first ever polymeric RO membranes in 1960 from CA. This membrane was capable of selective passage of water at a reasonable rate of flow under high pressure via blocking salts. In 1963, Loeb and Sourirajan [93] reported the use of an asymmetric CA membrane for desalination. However, the permeability of such early membranes were low, and RO membranes were better considered to be a novelty separation technique rather than desalination. Later, development of novel innovation in packaging of large membrane areas into small volumes as spiral wound modular configuration by General Atomics in 1963 opened the path for commercial applications. Such a spiral-wound configuration is now common in RO applications. Previously, membranes were either several-micron-thick polymer layers with a uniform architecture or similar-size polymer layers with an “asymmetric” structure with a nonporous salt-rejecting top surface opening up to a more porous support. In 1981, Cadotte patented the design of a three-layer TFC membrane of high permeability and, simultaneously, high water selectivity that is now the standard for industrial applications. 

PV is another membrane-based process, which has attracted increasing interest as a potential separation technique because of high selectivity and low energy consumption. The history of PV initiated in way back 1910, when Kober defined the term ‘per-vaporation’ via abbreviating ‘permeation’ and ‘evaporation’ after observing the selective permeation of water through the collodion and parchment membrane [94]. The process was first studied systematically to separate organic mixtures by Binning and co-workers at American Oil in the 1950s [95] and followed up in the 1970s by Aptel, Neel, and others [96]. By the 1980s, advances in membrane technology made it possible to prepare economically viable PV systems. The first commercial system for dehydration of azeotropic ethanol-water mixtures was installed by GFT in 1982. Since then, more than 100 plants have been installed for this application, of which the largest one was mounted in Bethenville, France, carrying the capacity of producing 5000 kg h^−1^ of ethanol [97]. The second commercial application of PV was the removal of traces of VOCs from contaminated water. This technology was developed by Membrane Technology and Research with the first commercial plant was installed in 1996 [98].

**Figure 2:**
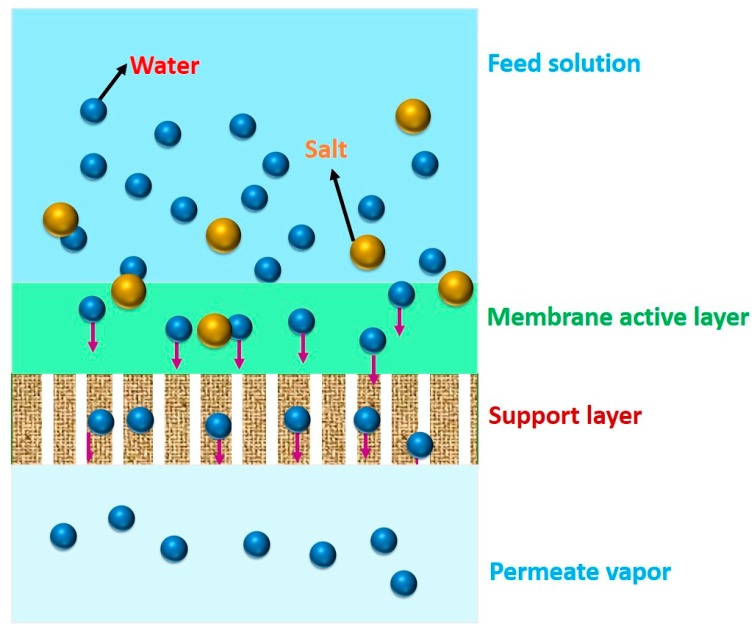
Desalination by the PV process where water passes through a dense PV membrane.

Desalination by PV is a combination of diffusion of water through a membrane, followed by evaporation into vapor phase on the other side of the membrane to produce fresh water (Figure 2). Although its application in desalination is rare, PV has been proved to be feasible and possesses few advantages for desalination, of which the primary advantage is significantly high salt rejection of monovalent salts (>99%) in PV [99,100]. The extent of salt rejection is independent of any variation in operating conditions. Issues, such as membrane wetting, salt leakage, and pore-plugging, are not serious in PV. The hydrophilic nature of PV membrane imparts beneficial desalination from feed solutions, along with the better anti-fouling property as compared to other membrane-based techniques, such as MD. Moreover, PV desalination does not require overcoming the osmotic pressure of the feed water. Thus, it can handle highly concentrated salt water without much adjustment in the driving force, which is related to ΔVP across the membrane. PV is projected as a feasible method in treating produced water from the mineral oil and natural gas extraction industries [101,102]. Pervaporative desalination is an efficient way of getting fresh water from non-potable saline sources with the advantage lying in its excellent salt rejection and capability of handling high-salinity solution. 

Although, PV possesses several advantages towards desalination, very few studies have been conducted in this field. So far, the best reported PV membranes are cellulose, organic-inorganic composite, silica, ionic polyethylene, and various polyether membranes, with the breakthrough water flux being reported for GO-PAN membrane. Such GO-PAN composite membranes have exhibited great potential for pervaporative desalination because of high flux of 3.62 kg m^−2^ h^−1^ and salt rejection of ~99.8% at 90 °C [50]. In recent years, there has been substantial interest in organic-inorganic hybrid membranes made of polymer matrix and inorganic NPs. Several kinds of inorganic NPs, such as SiO_2_, Al_2_O_3_, Fe_3_O_4_, ZnO, ZrO_2_, CdS, and TiO_2_, have been introduced into polymer matrices to prepare polymer-inorganic NP membranes. Inorganic particles have been dispersed in polymeric matrices for the preparation of dense or porous composite membranes carrying adaptable physical properties achieved during blending the properties of both organic polymers and inorganic dispersed particles. Polymer-clay hybrid NCPs have proven to be of immense interest because of the enhanced mechanical and thermal properties, reduced thermal expansion, gas permeability, and minimum swelling. There has been growing interest in discovering novel applications of porous carbon because of its ability to interact with molecules at their surfaces and within the bulk. PDMS-CMS, PVA-CNT, and PVA-graphite composites have been used for the removal of benzene from aqueous solution and separation of benzene-cyclohexane mixtures [103,104,105,106]. The development of a new type of polymer-inorganic hybrid membrane based on PVA-MA-silica for pervaporative desalination has been reported recently [46]. The hybrid membrane was synthesized via the sol-gel route using TEOS as the silica precursor with MA as an additional crosslinking agent. A water flux of 6.93 kg m^−2^ h^−1^ with salt rejection of >99.5% was achieved at 799.93 Pa vacuum and 22 °C. Another study reported the development of clinoptilolite-phosphate composite membrane for PV-based water desalination that showed high water fluxes of 15 kg m^−2^ h^−1^ and over 95% sodium removal in desalination of 1400 ppm saline water within 25–95 °C [107]. The future growth of the process will still be strongly dependent on the improvement of current membranes or development of novel membrane materials.

## 3. Polymeric Membranes for Pervaporative Desalination 

The use of polymeric membranes for pervaporative desalination is gaining high insight (Table 2). In the past, Korngold, Korin, and coworkers attempted desalination using polyethylene-based ion-exchange membranes [41,42]. Zwijnenberg et al. [43] studied pervaporative desalination using a tubular non-porous polyether amide membrane composed of ε-caprolactam and a mixture of poly(ethylene oxide) and poly(propylene oxide), bearing 40 μm thickness in a solar-driven PV chamber. Indeed, membranes based on polyether amide-based polymers were selected because of their higher swelling ability in water, accompanied by reasonably elevated permeability. Zwijnenberg et al. [43] showed that the pretreatment of highly-concentrated feed-water was not essential for the solar-driven pervaporation setup, as the deposition of fouling salts onto the membrane were almost negligible. Another polyether ester-based polymeric membrane developed by DuPont was utilized in pervaporative desalination of brackish- and contaminated-waters to obtain suitable water for irrigation [129]. Materials, such as polyester-based thermoplastic block copolymer and polyester supported cellulose triacetate membrane were employed as effective pervaporation membranes shoeing substantial flux and selectivity [101,130]. Again, cellulose containing the microstructures of densely packed polymer chains assembled via intermolecular H-bonds can potentially be used as membrane material for pervaporative desalination. Desalination performances of a series of hydrated cellulose derived from plant cellulose (i.e., wood or cotton), and bacterial cellulose membranes fabricated via surface cultivation of *Acetobacterxylinum* have been studied [131]. All of these membranes have demonstrated ~100% salt rejection at 40 °C at the feed concentration of 40 g L^−1^. Thereafter, Naim et al. [132] obtained the maximum flux of 5.97 kg m^−2^ h^−1^ for the pervaporative desalination of 40 g L^−1^ NaCl solution at 70 °C through a super-hydrophilic CA membrane synthesized via simple phase inversion method.

### 3.1. Synthesis

Various synthesis methodologies are available for preparing polymeric membranes suitable for pervaporative desalination, of which phase-inversion technique is the most widely used. For synthesizing cellulosic membrane via this method, CA powder was completely dissolved in the mixture of solvents containing acetone, dimethyl phthalate, and DMF. Then, the as-prepared solution was left for 2 days to remove air bubbles, so that the dried membrane will be devoid of defects incorporated through air bubbles. After that, the mixture was cast on a Petri dish for solvent evaporation, followed by immersion in ice-cold water, and finally, the membrane was deacetylated via alkali treatment for incorporating more O–H required for intermolecular H-bonding dependent film formation. 

### 3.2. Characterization

#### 3.2.1. Morphology

SEM micrograph of cellulosic membranes indicated the prevalence of asymmetric morphology showing pores of variegated sizes starting from superficial mini-pores at the skin layer to gradually enlarged pores at the bulk of the membrane. Such unique heterogeneous morphology played the pivotal role for elevating salt rejection efficiency. Interestingly, salt rejection ability is related to the presence of skinny layer bearing mini-pores, whereas higher flux was ascribed to the readily available porous structure of the remaining matrix. In this regard, crystalline deposition of organic matters, iron oxide, and other inorganic salts onto the pervaporative membrane [129], along with the associated membrane-fouling are usually investigated by the SEM analyses [43]. The crystalline deposition of characteristic cubic crystals of NaCl was noted to become scattered all over the surface of the membrane (Figure 6c from Sule 2013). In this regard, a fouling layer creates unwanted clogging at the membrane pores, leading to increased transport resistance against the flow of water.

#### 3.2.2. Mechanical Properties

Polymeric membranes suitable for pervaporative desalination should possess significant mechanical strength. In this context, the polyether ester-based polymeric membrane developed by DuPont was significantly endurable in terms of mechanical properties. 

#### 3.2.3. Spectroscopy

FTIR spectroscopy of dried cellulose membranes is essentially carried out to confirm the complete removal of unwanted solvents from membrane, incorporated during synthesis. Additionally, the prevalence of extra O–H introduced during deacetylation in cellulose membranes can be monitored through FTIR spectroscopy. In this regard, water affinity of cellulose and their transport properties are mostly dependent on the content of hydroxyl-groups and supramolecular organization, which are in turn governed usually by the origin of the cellulosic materials, along with modifications and drying techniques adopted at the time of membrane fabrication [131]. Accordingly, improved hydrophilic character of super-hydrophilic cellulosic membrane was understood from the appearance of three O–H specific *str.* peaks for each anhydro-glucose unit, coupled with physically adsorbed water-specific peaks within 1640–1630 cm^−1^ [132]. 

### 3.3. Desalination Performance and Mechanisms

#### 3.3.1. Membrane Structure

Membrane selectivity and mass transfer intensity through membrane are dependent on the physicochemical properties of the membrane-forming monomers and components of liquid mixtures. Both the elevated charge density in the range of 0.8–1.1 meq g^−1^ and decreased membrane thickness led to a higher water flux through the polyethylene membrane [41,42]. Moreover, charge characteristics of a membrane imparts a substantial influence on the flux and selectivity. Accordingly, it is expected that co-ions would pass through the membranes more readily than the counter-ions, as counter-ions can readily attach with the available opposite charges in the membrane. Additionally, steric factors, such as radius of hydration, may play a dominant role in determining the efficiency of membrane [101]. According to Xie et al. [140], the reduction in FFV of a membrane reduces the diffusivity and permeability of NaCl as well as the water uptake. In fact, because of the larger kinetic diameter, NaCl diffusivity is more sensitive towards the change in FFV than water diffusivity. In this regard, the hydrated sizes of Na^+^ and Cl^−^ are 0.72 nm and 0.66 nm, respectively, whereas the kinetic diameter of water is 0.27 nm [99,141,142]. Thus, selective separation of the desired component from a liquid mixture depends on the competitive diffusion of those components through the free volume units of a membrane. Theoretically, for attaining high selectivity, sizes of the component to be permeated should be comparable to the kinetic diameter of water molecules (i.e., 0.27 nm) [131]. Such a low free-volume dimension can only be achieved if the membranes contain structurally ordered segments at the supramolecular level. In case of plant cellulose (i.e., wood or cotton)-derived hydrated cellulose, the molecular architecture is composed of densely packed polymer chains, assembled via intermolecular H-bonds. In fact, relatively lower permeability of cellulose diacetate-treated wood cellulose membrane was attributed to the lower hydrophilic character of the cellulose diacetate components. Moreover, cotton cellulose-based membranes exhibited significantly higher permeability than wood cellulose-based membranes because of the variegated structural features of different celluloses. Furthermore, if the cellulosic membrane contains pores of variable sizes starting from minipores at the skinny layer to the larger pores at the bulk the higher flux and selectivity can be achieved [132]. Such low-cost membranes have been claimed to be more advantageous than some composite membranes prepared involving relatively complicated procedures [42,80]. 

#### 3.3.2. Influence of Feed Temperature

Temperature is one part of the overall driving forces producing movement of a permeant during the pervaporation process [143]. In this regard, the activation energy of permeation is often used to characterize the temperature dependence of permeation flux. As per Feng and Huang (1996) [144], the permeability of the membrane depends on both diffusivity and solubility of permeate. Since, both diffusion and solubility are temperature dependent, the activation energy of permeation should be expressed by the combination of activation energy of diffusion and enthalpy of dissolution. This is because the enthalpy change due to phase change in pervaporation influences the permeation behavior. 

Feed temperature has been found to influence strongly the flux and selectivity. With the increase in feed temperature, the total water flux was found to increase from 2.47 to 3.67 kg m^−2^ day^−1^ because of the enhanced molecular diffusivity and temperature-assisted reduction in water viscosity [137]. Such phenomenon was also visible for cellulosic membranes, since permeation and diffusion of gases or liquids across polymeric membranes were facilitated with the elevated temperatures [131,132]. However, permeability markedly decreased when the feed volume was not agitated for a significant period of time. Such lowering in permeability was termed as temperature-related polarization caused by regional drop in temperature at the membrane-water interface because of the change in aggregation of water into vapor [145]. Indeed, there is an intimate relationship among water flux, water temperature, and membrane thickness [101]. In general, increased membrane thickness deteriorates the water flux. However, as per Sule et al., water permeation rates remained almost the same when tubes of variable wall thicknesses (i.e., 0.5, 0.6, and 0.75 mm) were used as pervaporation membranes. Nevertheless, the permeate flux increases with increased difference in vapor pressures for membranes of varied thicknesses. However, an increase in flux is more pronounced when relatively thinner membranes are employed.

#### 3.3.3. Influence of Feed Concentration

In general, it is observed that water permeate flux decreases with the increase in solute concentrations [42,132,137,146] because of the increased concentration polarization adjacent to membrane surface. As expected, a reduced water uptake can be observed when concentrations of any of the salts, such as sodium chloride, magnesium chloride, and aluminum chloride, were increased [102]. It was observed that the sodium chloride tended to induce lower mass gains in the polyester membrane than those of magnesium chloride and aluminum chloride. In fact, the increased concentration of solute, such as sucrose and NaCl, can suppress the swelling of the membrane. Accordingly, the mass transport is affected because of the lesser availability of free volume inside the polymer matrix [42]. Moreover, the impact of enhanced feed concentration in reducing flux is mostly decided by the molecular size of the solute molecules. The presence of relatively bulkier molecules will block the pores of the membrane, leading to significant reduction in water flux. For instance, being larger in size, glucose molecules varying kinetic diameter = 0.86 nm block water flux more than that of NaCl, bearing a kinetic diameter of 0.30 nm [147]. Notably, minimal crystalline deposition onto the membrane after 96 h of uninterrupted pervaporation was actuated by the sweeping air flow at the outer surface of the membrane [137]. In fact, such sweeping air flow assists faster vaporization of moistures and, thus, creates an increased vapor pressure difference between the outer and inner surface of the membrane [101]. In this regard, fouling phenomenon is closely related to the increased feed concentration. Thus, both of these factors jointly contribute in lowering the driving force for transport of water through the membrane. However, the flux in solar-driven pervaporation setup was independent of feed concentrations and not affected by severe fouling resulted from the input of the concentrated feed [43]. 

## 4. Polymer Composite/NCP Membranes

Xie and co-workers [46,100] fabricated a series of hydrophilic PVA-MA-silica membranes for pervaporative desalination. Thereafter, Liang et al. reported the synthesis of GO-PAN composite membranes through a novel vacuum filtration-assisted assembly method which demonstrated high performance potential for pervaporative desalination [50]. Later, the same group fabricated a specialized three-layer composite membrane comprising of nonwoven PET, PVA, and nano-fibrous polyacrylonitrile (PAN) layers [80]. All the as-prepared multilayer composite membranes demonstrated excellent and consistent desalination proficiencies in terms of high water flux and salt rejection up to 99.5% for different feed concentrations even after 50 h of non-stop operation. Herein, the through-hole structures of nano-fibrous porous PAN layers encouraged faster evaporation of water passing through the topmost skin layer. Thus, this unique structure provided the possibility to overcome the defects of orthodox support layer prepared by phase inversion, leading to the enhancement of flux of the composite membranes. 

**Figure 3:**
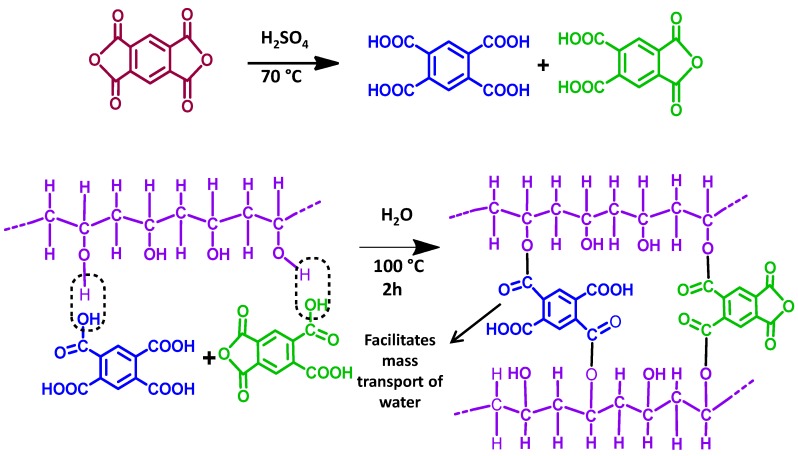
Crosslinking reaction during PMDA crosslinked PVA fabrication.

**Figure 4:**
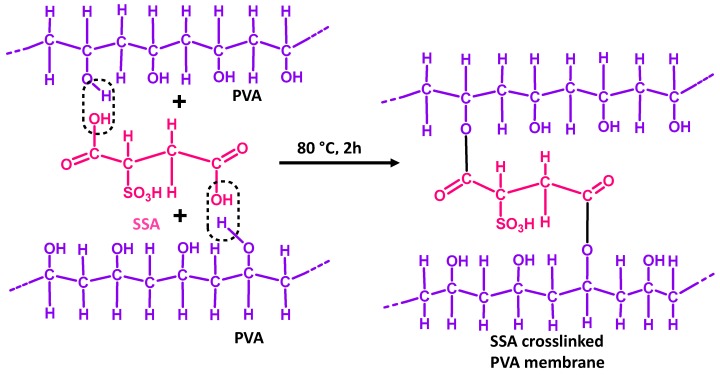
Crosslinking reaction during preparation of SSA crosslinked PVA membrane supported on PAN.

Again, the fabrication of a series of PVA-PAN-based composite membranes made of either PMDA or SSA crosslinked PVA coating on the top of the PAN UF membrane have also been reported [122,127]. The PAN support enhanced further mechanical stability to the PVA matrix crosslinked by hydrolyzed PMDA or SSA. Thus, ester-based crosslinks were formed via condensation reaction between O–H of PVA and –COOH of either hydrolyzed PMDA (Figure 3) or SSA (Figure 4). Moreover, the unreacted –COOH facilitated mass transport of water, while the phenyl ring of PMDA segments enhanced the FFV to actuate easier diffusion of water molecules. Accordingly, all PMDA-crosslinked PVA polymer demonstrated high water flux and fair stability in water. Among all of these membranes, the highest pervaporative desalination was achieved for composite membrane carrying 2 μm PVA selective layer crosslinked with 20 wt% of PMDA at 100 °C for 2 h. For this particular membrane, NaCl rejection was ~99.98% and water flux was 32.26 L m^−2^ h^−1^ at 70 °C and 35,000 ppm feed concentration. 

**Figure 5:**
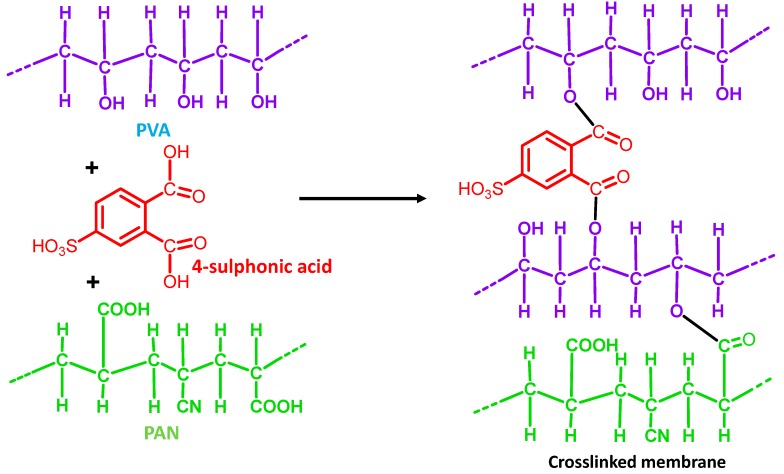
Crosslinking between alkali treated PAN and PVA by SPTA.

On the other hand, the best desalination performance was demonstrated by PVA-PAN composite membrane crosslinked by 18% SSA [122]. Recently, the same group tried to improve further the overall performance of PAN supported composite membranes via crosslinking PVA components by SPTA [128]. In these composite membranes, the sulfonic acid groups were strategically introduced to maintain the hydrophilicity and high flux of the membrane simultaneously, along with the desired crosslinking level to improve salt rejection (Figure 4 and Figure 5). 

Encouraging results were obtained while working with PEBA-based NCP membranes for desalinating high-salinity water [119]. For all the experiments conducted below 45 °C, ~99.9% salt rejection was obtained, along with the attainment of considerable permeate flux. Recently, Nigiz et al. designed zeolite 3A loaded PEBA membrane for pervaporative seawater desalination [120]. In fact, Zeolite 3A is a crystalline potassium aluminosilicate, which is derivated from zeolite 4A or sodium aluminosilicate. Introduction of zeolite 3A was mostly because of the incorporation of a certain degree of hydrophilicity to the hydrophobic PEBA matrix for successful improvement of water flux and salt rejection. Herein, the molecular sieving effect and H-bonding capability of water within zeolite 3A cages were utilized to increase water flux coupled with higher salt rejection. 

**Figure 6:**
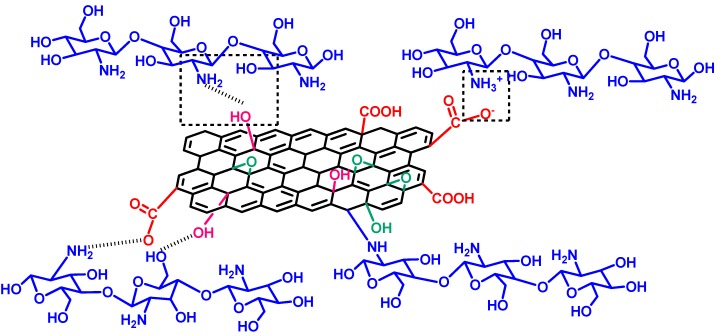
Covalently crosslinked graphene-CS membrane.

For further improvement of the desalination performance, GNPs have been incorporated in PEBA nanohybrid membranes [121]. In contrast to the earlier used traditional hydrophilic membranes, a hydrophobic PEBA matrix was utilized, along with the added GNPs to enhance the selectivity of membrane. Very recently, a high-performance novel GO-PI hollow fiber composite membrane was fabricated by direct spinning of GO nanosheet-PI suspension via coaxial two-capillary spinning strategy [126]. Another group of researchers prepared an efficient GO hybridized CS-based stable membrane for pervaporative desalination of high-salinity water [136]. In this regard, the possible interactions responsible for higher stability of membrane, such as reaction between epoxy groups of GO sheet and –NH_2_ of CS chains, and thereby formation of a crosslinked structure comprising of covalent bonds between GO and CS was explored (Figure 6). Other possibilities encompassing the ionic interaction between the –COO^−^ of GO and –NH_3_^+^ of CS, along with the strong H-bonding network in stabilizing the MMMs, was analyzed by extensive spectroscopic, thermal, and morphological characterization techniques. Thus, as-prepared highly efficient MMMs demonstrated improved mechanical stability because of fair chemical compatibility of GO with the CS matrix. 

Herein, similar to the O–H populated cellulose or modified cellulose membranes, composite membranes were fabricated based on O–H populated thin PVA membranes supported on PVS-based hollow fibers [114]. The idea behind the selection of such an assembly was to fabricate a mechanically reinforced membrane capable of faster and easier mass transport, while ensuring higher membrane surface area per molecule. In fact, hollow fibers containing PVS were selected as the supporting layer because of high mechanical strength, chemical resistance, and thermal stability. To improve the performance of the composite hollow membrane, PVS-made hollow fibers were coated with SiO_2_-incorporated PVA films [118]. Here, the main objective was to reinforce the top PVA layer of the composite membrane through crosslinking between SiO_2_ moieties and PVA chains, in which the silica precursor acted as both the crosslinker and inorganic filler.

In fact, such mechanical reinforcement was achieved by fabricating MMMs via incorporation of fillers, such as zeolites, (including silicalite-1 [148], ZSM-5 [149], and 4A zeolite [150], functionalized CNTs [151], and magnesium oxide [152]. Additionally, MMMs containing mesoporous inorganic materials, such as dual pore SBA-15 [153] and silica MCM-41 [154,155], have been proposed. Furthermore, MOFs were used to synthesize MMMs because of suitable size, shape, and chemical functionalities of the MOF cavities [156,157]. In this context, Kang et al. synthesized MMM using ZIFs, mainly ZIF-7 embedded in a CS polymer matrix with 5 wt% MOF loading, for pervaporative removals of water-ethanol mixtures, exhibiting almost a 19 times higher separation factor than that of pure polymer [158]. Hua et al. reported ZIF-90-PI P84 MMMs for the dehydration of isopropanol [159]. Again, Kasik and Lin reported the organoselective MMM carrying MOF-5-filler [160]. For ZIF-8-based MMMs, the PV performance of silicone rubber membranes was improved significantly for isobutanol and furfural recovery from their aqueous solutions via introducing ZIF-8 NPs [161,162]. Sorribas et al. reported the use of MMM containing MOF Cu_3_(BTC)_2_ (i.e., HKUST-1) within the commercial PI Matrimid^®^5218 as polymer matrix for pervaporative separation of water-ethanol separation with improved water flux [163]. 

In order to overcome the problems associated with the existence of micro-defects in PVA membrane, variegated procedures were adopted to achieve the best possible solution regarding fabrication of defect-free membranes. In this context, micro-defects of the PVA-PVDF-based composite membrane were explored by comparing the different coating methods, such as dip-coating and cast coating [164], along with the enhancement of the storage stability. Another PVA-like polymer material, i.e., PEBAX, was utilized for fabrication of PV membrane, since PEBAX is similar to PVA with respect to the properties, such as high hydrophilicity and water vapor permeability [165]. Instead of high water vapor permeability, PEBAX-based membranes are not suitable for desalination because of high free volume and flexible rubbery sections, which unselectively transport both water molecules and hydrated salt ions. At lower feed concentration and at higher temperature, sufficient water flux was observed for PEBAX membranes. However, when the NaCl concentration was increased up to 100 g L^−1^, flux was found to reduce drastically to 2 L m^−2^ h^−1^. Later, for the first time, Yilman et al. reported the fabrication of MWCNT doped PVA membrane for pervaporative desalination [100]. 

### 4.1. Synthesis

#### 4.1.1. Sol-Gel Method

**Figure 7:**
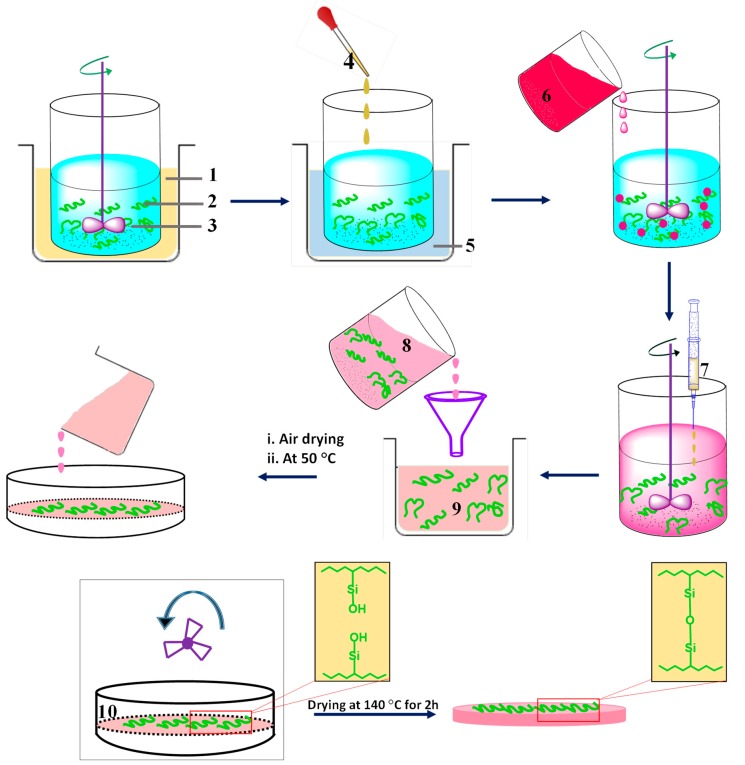
Sol-gel method for preparing PV-membrane: 1. PVA Chains, 2. Silicone oil bath at 90 C, 3. Water, 4. Cold water, 5. Concentrated HCl, 6. MA, 7. TEOS-ethanol mixture, 8. Reaction mixture, 9. Filtrate, 10. Membrane film.

Various workers reported the synthesis of polymeric membranes through sol-gel method [46,100]. Xie et al. synthesized PVA-MA-silica hybrid membranes using an aqueous route, wherein TEOS and MA were used as silica precursor and additional crosslinker, respectively (Figure 7). Initially, PVA (>98% hydrolyzed and molecular weight = 130,000 g mol^−1^) was dissolved in 100 mL deionized water in a silicone oil bath at 90 °C to obtain 0.75 wt% PVA solution, followed by cooling and pH adjustment at 1.7 ± 0.1 by dropwise addition of ~0.5 mL concentrated HCl (Figure 7). Then 0.15 g of MA (weight content of MA with respect to PVA = 20 wt%) was added to as-prepared PVA solution and stirred till dissolved completely. In the next step, TEOS and ethanol mixture bearing mass ratio of TEOS:ethanol = 1:9 was added dropwise to the above solution with constant stirring. The amount of TEOS should be added to such extent that the weight content of SiO_2_ with respect to the amount of PVA in the solution should be varied within 10–25 wt%. Thereafter, the reaction was continued at room temperature in acidic environment for 2 h, and the resultant homogeneous mixture was filtered and casted on Petri dishes to attain the desired thickness, followed by air drying. The reaction was carried out in acidic environment for ensuring faster hydrolysis of TEOS rather than condensation of Si–OH bonds, so that frequent amount of Si–OH groups are allocated for obtaining linear or random branches of silica network [166]. Finally, the obtained film was dried further at 50 °C overnight and heated to 140 °C for 2 h in a fan-forced oven. The main objective of such higher temperature drying was to produce siloxane bonds through condensation reactions among silanol groups [167,168].

#### 4.1.2. Solution Mixing and Casting

**Figure 8:**
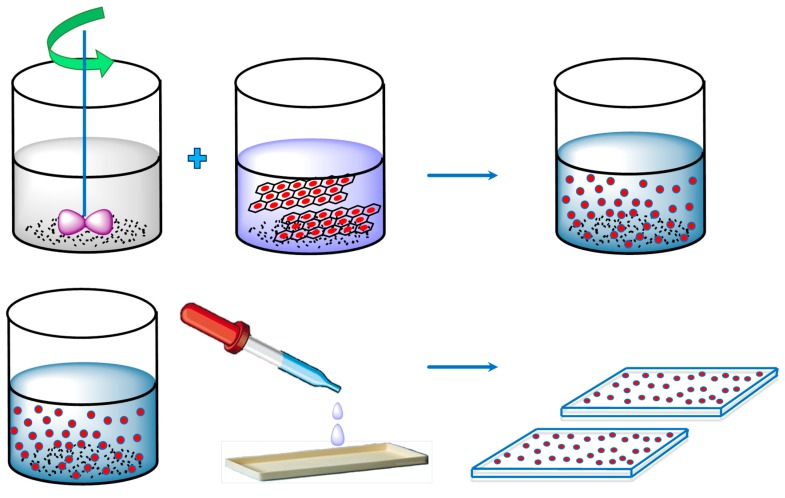
Preparation of membrane by solution mixing and casting.

Few researchers synthesized PEBA-GNP membranes, bearing different quantities of GNPs, using the phase inversion technique (Figure 8) [121]. Initially, 10 wt% homogeneous PEBA solution was prepared in acetic acid by constant stirring for 5 h at 60 °C. In another setup, GNPs were dispersed in acetic acid by continuous mixing for 15 min in an ultrasonicator. Thereafter, the solution of PEBA was admixed to the dispersion of acetic acid-GNPs with constant stirring for another 2 h at room temperature, followed by solution casting on a Teflon plate to obtain flat membranes of uniform thickness (i.e., 120 ± 5 μm). 

Similar technique was also adopted for fabricating MWCNT-doped PVA membranes [100]. Herein, GA was added as crosslinker in MWCNT containing PVA dispersion casted on PMMA surface. Similar to the technique used for fabricating PEBA-GNP membranes [121], PEBA, acetic acid, zeolite 3A were used to prepare all the requisite pre-membrane solutions during preparation of zeolite 3A-filled PEBA membrane, followed by rigorous homogenization, admixing, and casting on a Teflon sheet [120]. The same technique was also adopted to produce different PEBA-based composite membranes containing suitable combinations of porous components, such as PAN, PE, PSF, and sodium NPs (NaX) [119].

GO-incorporated CS-based MMMs were fabricated by such a popular technique [136], after the usual preparation of GO-nanosheets by the modified Hummers method [169]. While preparing the PVA-PAN composite membrane [127], initially, a PMDA-crosslinked dense film of PVA was prepared by solution-casting, followed by preparation of composite membrane by the dip-coating method. In this step, an alkali-treated hydrolyzed PAN membrane was dipped into a dope solution of PVA for 30 s, followed by the PVA-coated PAN membrane air drying for 48 h at room temperature, and subsequent heating to initiate crosslinking. During hydrolysis of PAN, the –CN were converted to –COOH, which actively interacted with O–H of PVA for improving adhesion property of PAN [170]. In this regard, optimum flux and separation performance were achieved when crosslinking temperature was maintained at 100 °C. 

**Figure 9:**
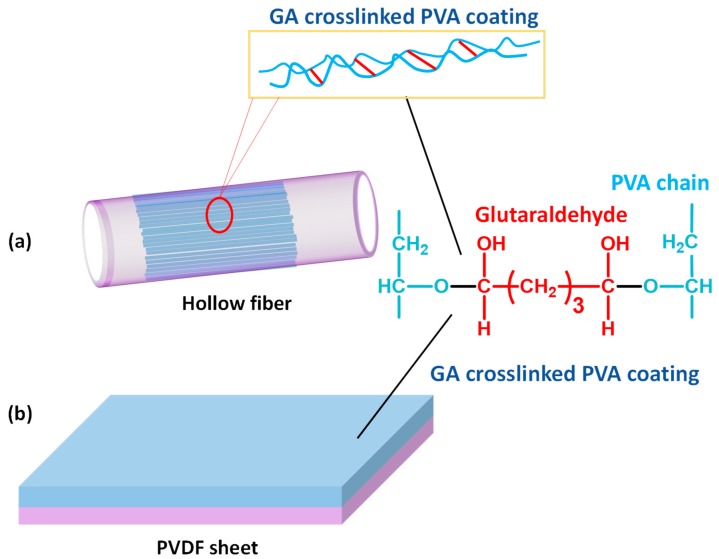
GA crosslinked PVA-coated PVDF membranes prepared by (**a**) dip coating and (**b**) cast coating.

In order to compare the pervaporative desalinating efficiencies of GA crosslinked PVA membranes supported on PVDF, two types PVA coated PVDF membranes were prepared individually by dip- and cast-coating methods [123]. Of these, one set of composite membranes were prepared by dip-coating of PVDF hollow fibers, ensuring only single-side coating of hollow fibers by sealing the hollow fiber membrane prior to dip-coating (Figure 9a). Another set of PVA-PVDF membranes were prepared by coating the crosslinked PVA layer on the flat sheets of PVDF membranes (Figure 9b). While preparing the dip-coated PVA-PVDF membranes, dried and sealed hollow fibers of PVDFs were immersed in the previously prepared coating solution of variable PVA and GA concentrations, followed by soaking for stipulated time period. In this context, the molar ratio of GA and PVA was maintained consistently at 0.2, so that the crosslinked structure of the final PVA coated layer remains unaltered. Subsequently, the soaked hollow fibers were dried at 50 °C for 24 h to actuate crosslinking among all the individual components. While preparing the same composite by cast-coating, casting of crosslinked PVA was conducted on flat sheets of PVDF by casting machine operated at 50 mm s^−1^ under 50% humidity. In the next step, the PVA coated membranes were dried at room temperature overnight, followed by oven drying for 30 min at 80 °C.

#### 4.1.3. Vacuum Filtration-Assisted Assembly Method

**Figure 10:**
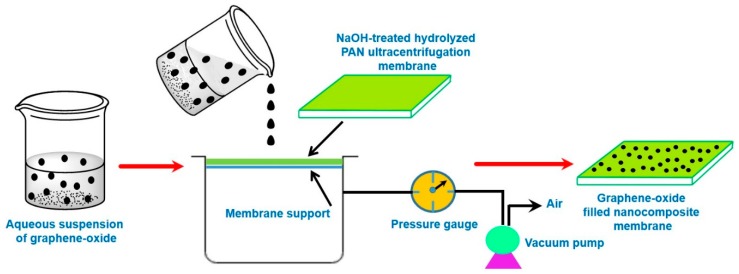
Vacuum filtration assisted assembly method.

Additional attempts were devoted to prepare functionalized GO-deposited PAN membranes via the vacuum filtration-assisted UF method [50]. Initially, an aqueous suspension of GO was prepared by the modified Hummers’ method [170,171]. Thereafter, GO-PAN composite membranes were prepared by vacuum filtration of the well dispersed aqueous GO suspensions through NaOH treated hydrolyzed PAN UF-membrane (Figure 10). In this regard, further homogenization of the aqueous GO suspension was carried out to ensure the minimum aggregation. Accordingly, functionalized GO residues were deposited and distributed throughout the PAN-UF membrane to fabricate GO-filled composite membranes.

#### 4.1.4. Direct Spinning and Phase Inversion Method

**Figure 11:**
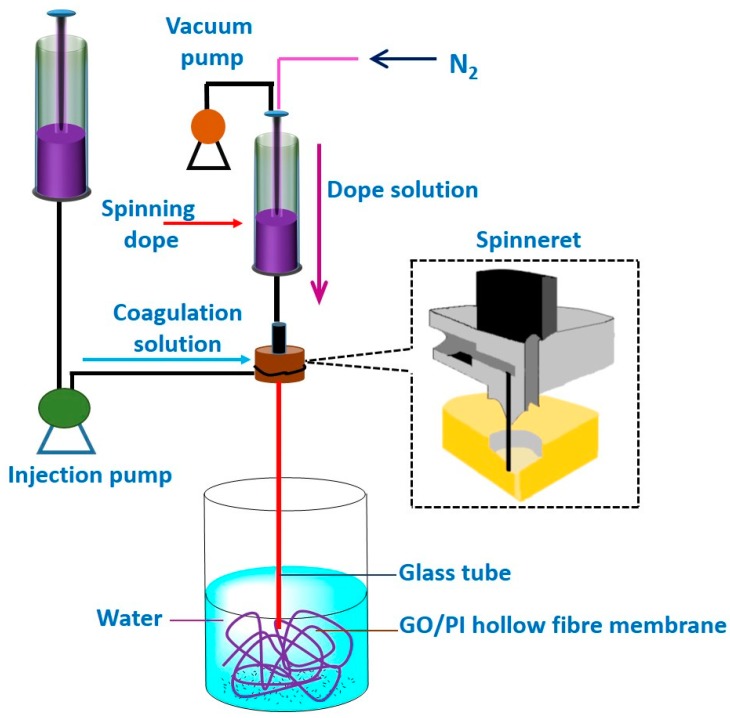
Schematic diagram of preparation of GO/PI hollow fiber membranes by direct spinning of a GO/PI suspension via phase inversion in water/NMP solution.

Direct spinning coupled with phase inversion technique was used to prepare GO- PI hollow fiber membranes suitable for efficient pervaporative desalination of salty water (Figure 11) [126]. Initially, aqueous suspensions of GO were prepared by the modified Hummers’ method [116,171]. Thereafter, GO powder was produced after necessary purification of GO suspension, followed by freeze drying of the purified suspension. Later, the asymmetric GO-PI hollow fiber membranes were prepared by direct spinning of a GO-PI suspension via phase inversion in presence of water-NMP coagulation.

A similar method was adopted by Chaudhri et al. to prepare composite PVA membranes supported on PVS-based hollow fibers [114]. In this two-step method, initially, PVS-based hollow fibers were prepared by spinning through a double orifice spinneret from a transparent spinning dope composition of 20% (*w*/*w*) PSF, 2% (*w*/*w*) polyvinylpyrrolidone, and 78% (*w*/*w*) DMF under N_2_ environment, followed by automatic coating and crosslinking by PVA and MA, respectively [114]. To further enhance the efficiency of the membrane, SiO_2_ incorporated composite PVA membranes were fabricated via coating the PVS-based hollow fibers with a different coating solution comprising of aged PVA-SiO_2_ sol in acidified and hydrated ethanol [118]. In fact, during preparation of the coating solution, the employed strategy was pretty much similar to the strategy adopted by Xie et al. [46,100], while crosslinking the membranes by TEOS in acidic environment. 

A multi-step procedure was followed to achieve PVA-SiO_2_ coated composite hollow membrane. Herein, after preparing the acidified aqueous PVA and alcoholic TEOS solutions, TEOS solution was added dropwise and admixed to the PVA solution, so that a constant ratio of PVA:TEOS:HCl = 1:1:2.14 was maintained in the mixture. In this context, the water:ethanol was maintained more than two to avoid immiscibility during addition of alcoholic TEOS in acidified PVA. In fact, such a higher amount of water also prevents unwanted self-condensation reaction of the silicate groups of hydrolyzed TEOS resulting in the high-dimensional spherical particle structure. In this way, the silicate groups generated from hydrolyzed TEOS get ample opportunity to react with the PVA chains to form the coating solution. Finally, the solution was undergone aging for 36 h for producing coating solution, which was subsequently applied for coating on the PVS-based hollow fiber. Thereafter, the coated fiber was dried at room temperature to evaporate and remove solvents to achieve sufficient hydrolysis of TEOS and thereby production of Si-OH, which interacts with O–H of PVA to generate ether linkages during drying and degassing [118]. 

#### 4.1.5. Electrospraying and Electrospinning

The above mentioned combined method was also utilized for synthesizing multilayer composite membranes comprising of PVA, PAN, and PET materials [80]. At first, PVA base layer was electrosprayed onto the surface of Al-foil, followed by deposition of the nanofibrous mid-layer of PAN on the PVA base layer via electrospinning. Herein, the major function of nanofibrous PAN layer was to provide the desired physico-chemical stability and mechanical strength. Thereafter, a nonwoven PET layer was attached at the top to produce the assembled composite membrane. PVA components of some of these assembled composite membranes were intentionally crosslinked by treating them with GA-based crosslinking solution. Such crosslinking of PVA was carried out to minimize the swelling and dissolving tendency of PVA in water, which often restricts the use of PVA in water treatment applications [172,173]. Another objective behind such crosslinking was to restrict the transportation of larger hydrated ions. 

### 4.2. Characterization

#### 4.2.1. Morphology

**Figure 12:**
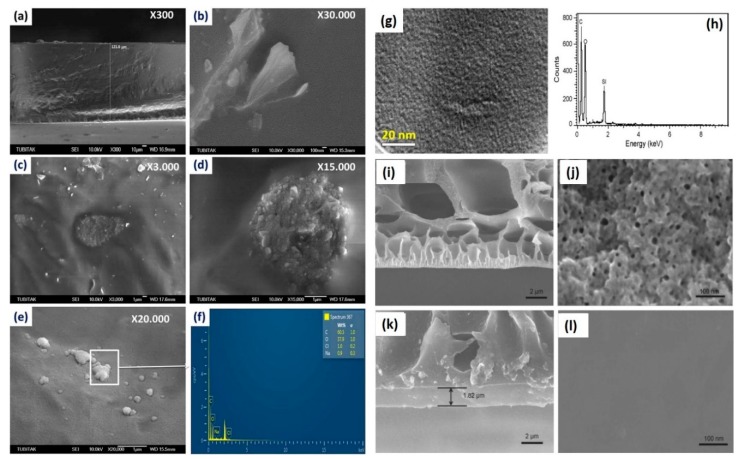
Cross-sectional SEM micrographs of the (**a**) pristine, (**b**) 2 wt% GNPs-PEBA, and (**c**,**d**) 5 wt% GNPs-PEBA; (**e**) surface micrograph of 2 wt% GNPs-PEBA (after desalination); and (**f**) EDX result of 2 wt% GNPs-PEBA membranes. Reprinted with permission from [121]. Copyright 2017 Elsevier B.V. (**g**) TEM image and (**h**) EDS spectra of the hybrid PVA-MA-silica membrane containing 20 wt% MA and 10 wt% SiO_2_ with respect to PVA. Reprinted with permission from [46]. Copyright 2011 Elsevier B.V. SEM images of (**i**) cross-section and (**j**) top surface of the PAN ultrafiltration membrane and of (**k**) cross-section and (**l**) surface of PVA-PAN composite membranes [127].

Within PEBA-GNP membranes (Figure 12a–f), GNPs were noted to become intercalated in the PEBA matrix, and the agglomerates of GNPs were observed in the NCPs bearing higher concentration of GNPs [121]. However, such agglomerates are not desirable for achieving the optimum performance of membranes [120]. In this context, both the bulk and surface of MWCNT-loaded PVA membrane were not free from such agglomerations [100], especially in MWCNT-PVA composites possessing higher amount of filler. However, for hybrid PVA-MA-silica membrane, no larger particulate agglomerates (>10 nm) were observed in TEM images (Figure 12g), suggesting uniform dispersion and distribution of silica NPs within PVA matrix.

The intimate polymer-filler interaction and uniform distribution of silica NPs resulted in partial destruction of inherent crystallinity of PVA matrix [100], as apprehended from the appreciable broadening and weakening of PVA-specific XRD peak at 20° of hybrid PVA-MA-silica membrane [174]. Indeed, such increased amorphous characteristics could also be visualized from clear transparent nature of the membrane in contrast to the brown-colored opaque appearance of PVA-silica membrane. Crosslinking-driven destruction of crystallinity for PVA was also manifested in the GA-treated PVA [80]. Additionally, crystallinity of PVA was noted to deteriorate when PVA layer was electrosprayed on the Al-foil, associated with quicker evaporation of the volatile solvent of PVA layer, followed by rapid solidification. 

While studying the composite PVA membranes supported on PVS-based hollow fibers, it was observed that the hollow fibers are not exactly circular since the extruded fibers experienced considerable mechanical stretching during winding [114]. Moreover, the hollow fiber structure was asymmetric in nature and were comprised of smaller pores near the surface and larger macropores in the interior, similar to the pore characteristics of the asymmetric cellulosic membranes fabricated by Naim et al. [132]. Herein, the smaller pores were the active pores, responsible for separation of unwanted species. 

The morphological characteristics of PAN-PVA membranes (Figure 12i–l) [127] were composed of large finger-like micro-voids in the PAN portion underneath the skin layer of PVA. Such micro-voids were crucial in reducing the mass-transfer resistance against the water vapor during pervaporative desalination. Moreover, small pores of nano-dimensions were located at the top surface of the PAN membrane, which were generated because of alkaline hydrolysis of the PAN membrane during dip-coating. These pores contributed significantly in the permeation of water vapor during pervaporative desalination process. Interestingly, the mean pore size and the surface porosity of PAN membrane increased dramatically when hydrolysis time was increased from 1 to 2 h. Such an abrupt increase in the mean pore size from 6.9 to 187.4 nm was associated with the rupture of PAN polymer chains during alkaline hydrolysis [128]. 

**Figure 13:**
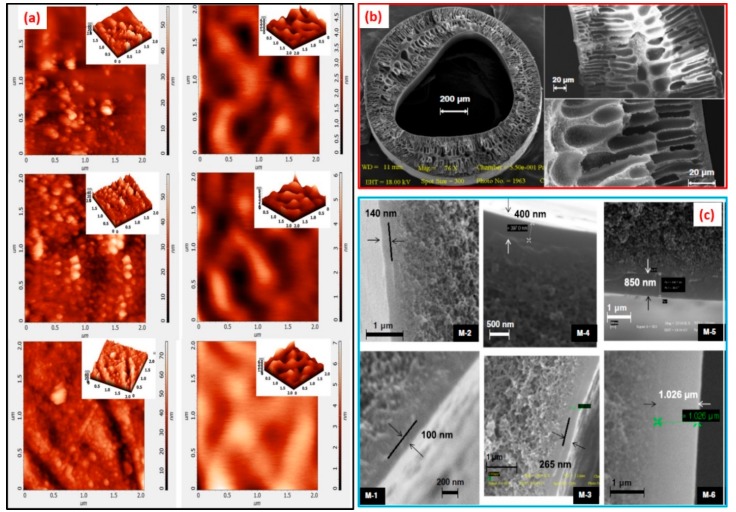
Cross-sectional SEM images of (**a**) hollow fiber support; (**b**) composite membranes; and (**c**) top surface AFM images of the composite membranes and (inset) 3D images of the respective samples. Reprinted with permission from [114]. Copyright 2015 Elsevier B.V.

**Figure 14:**
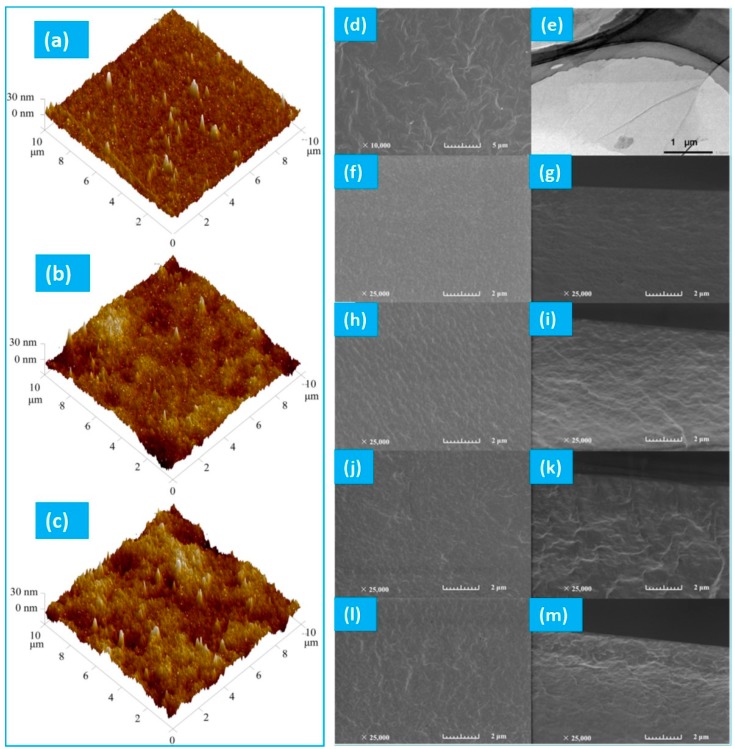
AFM images of (**a**) pristine CS membrane, (**b**) CS/GO-1, and (**c**) CS/GO-2 MMMs. (**d**) SEM and (**e**) TEM of GO and SEM images of the top surface and cross-section of (**f**,**g**) CS, (**h**,**i**) CS/GO-0.5, (**j**,**k**) CS/GO-1.5, and (**l**,**m**) CS/GO-2 membranes. Reprinted with permission from [136]. Copyright 2018 Elsevier B.V.

AFM characterization was sought to monitor the change in topological details for a membrane undergoing pervaporative desalination. In this context, the surface topology of composite PVA membrane was analyzed from the respective AFM images (Figure 13a and Figure 14a–c) [114]. It was observed that the surface roughness was decreased with an increase in coating layer thickness. In case of GO-hybridized CS membranes (Figure 14) [136], enhanced roughening was observed with addition of higher amounts of GO that was attributed to the exposure of GO flakes onto the membrane surface consisting of ridges and valleys [175]. Additionally, the cross-section of the membrane envisaged enhanced roughness with greater population of GO sheets in the hybridized membrane (Figure 14). The increase in roughness onto hydrophilic surface could result in the increased wettability of water onto the membrane surface favoring improved water permeation through the membrane. 

**Figure 15:**
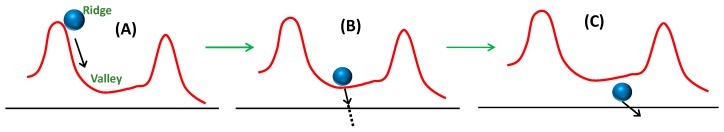
Accelerated water transport on rough membrane comprising of ridge and valley.

In this regard, water tends to flow preferentially from the ridges to the valleys that effectively produce a shorter diffusion path length and thereby generating higher flux (Figure 15). Moreover, almost vertical orientation of GO sheets at the membrane surface is desired to achieve accelerated flux. However, while running the pervaporative separation, foulants may be trapped and accumulated in the valleys, resulting in clogging of the pathway despite accelerated flow from ridge to valley. For estimation of the possible incorporation of SiO_2_ in PVA-SiO_2_-based composite membrane, Chaudhri et al. compared the thickness of the coated composite membranes from the respective SEM photomicrographs (Figure 13b,c) and by employing a gravimetric method based on Equation (1) [118]:

(1) $$t=\frac{W_{c}-W_{u}}{d\times A}$$

Here, *t*, *W_c_*/*W_u_*, *d*, and *A* are thickness, weights of the coated/uncoated fibers, density, and surface area of fiber, respectively. The *A* is calculated from Equation (2), in which *r* and *l* are outer radius and length of the fiber, respectively:

(2) A=2πrl

The main objective behind evaluating thicknesses by both SEM and gravimetric analyses was to detect the incorporation of SiO_2_ in the top layer and hollow fiber portions. It was observed that the gravimetric equation-derived thickness was almost twice as compared to the SEM-derived thickness because of the huge difference between *W_c_* and *W_u_* as a result of incorporating a significant amount of SiO_2_ within PVA-SiO_2_ film. In this regard, distribution of the incorporated SiO_2_ was also evaluated via EDX analyses (Figure 12h), which demonstrated a very large population of Si at the surface of the top layer, whereas the inner layer possessed lower population Si, suggesting intrusion of SiO_2_ at the inner layer of the composite membrane. 

In GO-PAN composite membranes, the vacuum assisted filtration-based technique resulted in exfoliated distribution of GO particles in PAN membrane [50], suggesting formation of substantial interfacial area between PAN and GO. Indeed, agglomeration of GO particle could be avoided in GO-PAN composite membranes because of the sufficient homogenization of GO suspension during preparation of the composite membrane. In addition, a high-performance PV membrane should also be devoid of defects, such as cracks and pinholes [126]. 

The lateral pore structures contributed significantly in enhancing the water fluxes of the asymmetric GO-PI hollow fiber membranes. Additionally, the pore characteristics of such membranes influenced to achieve higher water flux and improved membrane selectivity than PI-based hollow fiber membranes containing frequent macropores. In this regard, though finger- and sponge-like pores were much denser in PI-based hollow fiber membrane than GO-PI hollow fiber membranes, the BET-specific surface area of GO-PI hollow fiber membranes were found to be much higher than PI-based hollow fiber membranes. Thus, the incorporated GO played a crucial role in forming porous membranes. 

**Figure 16:**
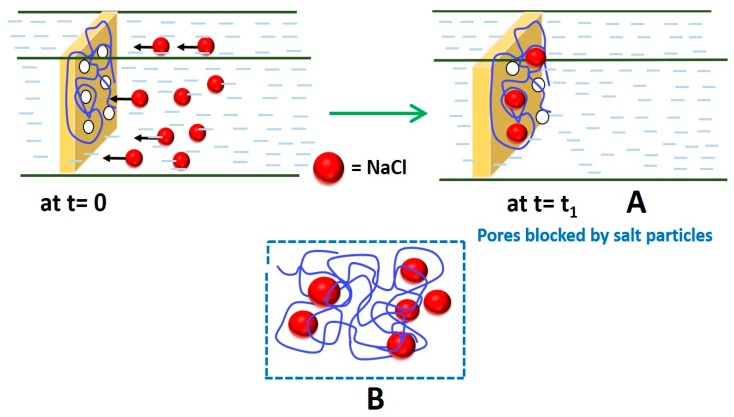
Time dependent deterioration of flux by (**A**) blocking of pores by crystalline deposition of salt and (**B**) occupation of free volume by hydrated salt species.

In this regard, the hydrophilic groups in GO accelerated the rate of solvent and non-solvent exchange during phase-inversion process [176]. Influence of pore characteristics and micro-defects can contribute significantly in the performance of PVA-PVDF composite membranes [123]. In this context, pore size and structure of PVDF can be altered depending on the PVA concentration in dipping solution. During the dip-coating process, PVA solution of low concentration and viscosity can penetrate easily into the pores of PVDF hollow membrane, leading to the insufficient thickness of the coating layer at the PVDF surface. On the other hand, highly concentrated and viscous PVA solution can produce the undesirably thicker coating layer and thus reduced flux. Thus, both of these aspects are unfavorable in fabricating a thin PVA selective layer equipped with desirable PV efficiency. Moreover, time-dependent deterioration of flux was observed, irrespective of the thickness of PVA layer. Such phenomenon was observed because of the superficial deposition of crystalline particulate matters of salt on the permeate side of the membrane [123], along with the hydrated salt ions occupying free volume within PVA matrix, blocking the passage of water molecules (Figure 16). 

#### 4.2.2. FTIR Analyses

FTIR analyses were carried out to identify the formation or destruction of bonds during synthesis of the membranes. By means of FTIR analyses, reactions and thereby intimate association among various components of the hybrid PVA-MA-silica membrane was realized because of the formation of newly generated peaks. The FTIR spectrum of the PVA-MA-silica membrane showed an ester-specific new peak at 1726 cm^−1^, as a result of grafting or crosslinking via esterification reaction between O–H of PVA and –COOH of MA [46]. Another new peak at 950 cm^−1^ was attributed to Si–OH resulting from hydrolysis of TEOS and associated H-bonds between organic groups and silica. Moreover, significant rise in the peak intensity at 1080 cm^−1^ was associated with the formation of Si–O–Si via condensation reaction between hydrolyzed silanol-specific Si–OH and crosslinking between PVA and TEOS. 

Chaudhri et al. also observed similar types of reactions during monitoring the time-dependent change in FTIR spectra during aging of TEOS treated PVA-SiO_2_ film, to be used as the top layer of the polyvinylpyrolidone hollow fiber supported composite membrane [118]. Interestingly, with the increase in aging time from 24 to 72 h, the peaks within 1000–1200 cm^−1^ were found to broaden because of the contribution of newly generated C–O–H bending and Si-O-Si and Si-O-C asym. str. Arrival of such vibrations can only be possible because of hydrolysis and self-condensation of Si–OH or condensation of Si–OH and O–H of PVA. Indeed, the involvement of O–H of PVA during crosslinking was also detected from the complete disappearance of PVA-specific peak at 1583 cm^−1^ assigned to C–O of C–OH of PVA. Moreover, the existence of Si–OH in PVA-SiO_2_ was also substantiated from the prevalence of a broad shoulder at 970 cm^−1^, interrelated with the increased hydrophilicity of composite membrane. Indeed, such a reaction between PVA and SiO_2_ also resulted in the destruction of the crystallinity in the PVA-SiO_2_ membrane, as reported earlier by Xie et al. [100]. 

**Figure 17:**
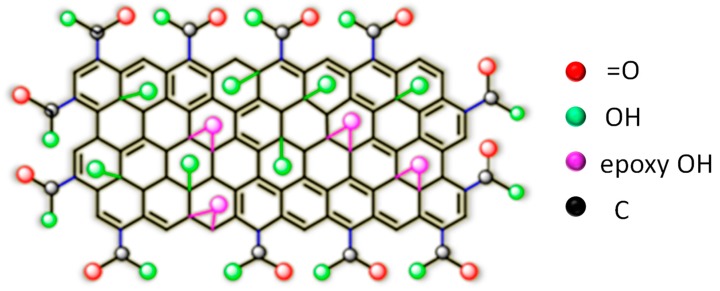
Lerf–Klinowski model of GO.

For GO-PAN composite membranes, strong H-bonding among diversified functional groups of GO, such as C–O, C=O, and O–C=O with –COOH and –CONH_2_ of PAN [177] was observed. Indeed, the existence of these oxidation-derived functional groups in GO, conforming to the Lerf–Klinowski Model (Figure 17) [140], was rationalized by FTIR, XPS, and Raman spectroscopy [50]. 

In another report, the reaction of O–H of GO with PI and thereby formation of ether linkage in GO-PI hollow fiber membranes was realized from FTIR analyses [126]. Similarly, crosslinking of O–H of PVA with MA in composite PVA membranes supported on PVS-based hollow fibers was realized from the loss of PVA-specific free O–H and –COOH of MA [114]. Condensation of –COOH of PMDA crosslinker and O–H of PVA in preparing the PVA-PAN composite membrane was detected from the continuous loss of intensity for O–H specific broad peak centered at 3320 cm^−1^ [178,179,180,181,182,183], along with the consequent gain in C=O and –C–O–C– peaks of aromatic ester at 1750 and 1275 cm^−1^, respectively [127]. 

Similarly, for SSA crosslinked PVA film, the peaks at 1030 cm^−1^ and 1226 cm^−1^ represent asym and sym str. S=O of the sulfonic acid group, respectively [122,184,185]. For GO hybridized CS membrane, reaction between primary –NH_2_ of CS with epoxy group of GO was identified from the increase in N–H bending, along with the weakening of primary –NH_2_ peak at 1651 cm^−1^ [136,186,187,188]. Though, the authors have not reported the change in FTIR peak corresponding to the epoxy group of GO as a result of reaction with primary –NH_2_, such reaction was confirmed from the increased intensity of C–N specific deconvoluted C1s spectra for the GO hybridized membrane. Moreover, by monitoring the change in N1s XPS spectra, the authors reported ionic interaction of –NH_3_^+^ of CS with –COO^−^ of GO moieties. Additionally, strong H-bonding interactions between GO and CS was also realized from the FTIR and XPS analyses.

#### 4.2.3. Thermal Properties

Noticeable changes were observed in the characteristic transition peaks of DSC thermogram for PEBA block copolymer comprising of soft polyether and hard polyamide segments [121]. As a result of GNP addition in PEBA, absolute disappearance of polyether-specific peak at 58 °C was associated with the interaction of GNPs with polyether moieties, resulting in the enhanced thermal stability and reduced crystallinity of PEBA-GNP NCP membranes than the unfilled PEBA matrices.

The enhanced thermal stability was also noticed for hybrid PVA-MA-silica membranes as compared to the membranes devoid of MA crosslinking at high temperature, substantiating the MA-assisted crosslinking in hybrid PVA-MA-silica membranes. MA- and silica-mediated crosslinking dependent improvement in thermal stability was also exemplified from the continuous rise of the characteristic T_g_ of PVA from 84 to 94 and 103 °C for PVA-silica and PVA-MA-silica membranes, respectively [46]. With the rise in MA content, similar increase in T_g_ from 93 to 136 °C was also observed in composite PVA membranes supported on PVS-based hollow fibers because of increased crosslinking between O–H of PVA and –COOH of MA [114]. Such crosslinking-driven loss of crystallinity in composite membranes was also realized from the considerable decrease in endothermic melting peaks at 220–340 °C of the polymer.

Crosslinking and associated improvement in thermal stability of PVA-SiO_2_ over PVA was realized while comparing the respective TGA plots [118], along with the appreciable enhancement in the melting point of PVA-SiO_2_ at 236 °C as compared to pure PVA at 217 °C. In an another study, the crosslinking of PVA by GA was realized from the comparative TGA plots showing higher thermal stability for GA crosslinked PVA membrane over untreated PVA [80]. Indeed, crosslinking was substantiated from the disappearance of the characteristic endothermic peak of pure PVA at 225 °C [189,190].

In this regard, crosslinking of PVA by PMDA was realized from the enhanced thermal stability of PMDA crosslinked PVA over pure PVA within 120–250 °C, suggesting crosslinking-originated retarded decomposition of PVA side chains in PMDA crosslinked PVA [127,191]. Invariably, better thermal stability in lower temperature range was observed for SSA crosslinked PVA over uncrosslinked PVA [122].

The poor thermal stability of SSA crosslinked PVA at high temperature region was attributed to the de-sulfonation and dissociation of ester linkages [192]. Moreover, higher crosslinking resulted in the enhancement of T_g_ as well as the disappearance of melting temperature in SSA crosslinked PVA, which indicated the restricted formation of crystalline structure in PVA polymer together with the increased polymer chain rigidity [193]. As a result of H-bonding and other covalent/non-covalent interactions between individual components [194,195], such restricted movement of the polymer chains was noticed in the GO hybridized CS membrane, which was reflected from the increased T_g_ of the GO hybridized CS over the mere CS membrane [136].

#### 4.2.4. Mechanical Properties

Due to the reaction between O–H of GO and PI, newly formed ethereal crosslinks were responsible for the enhanced tensile modulus of GO-PI hollow fiber membranes over PI hollow fiber membranes [126]. However, the TS and EAB of the GO-PI hollow fiber membrane were less than the PI hollow fiber membranes, since the mobility of polymer chains was restricted because of the formation of crosslinks [196,197]. For GO-hybridized CS membrane, appreciable increase in both TS and EAB was observed in 0.5 wt% GO-loaded CS membrane [136]. However, with the further increase in GO content, TS was found to deteriorate because of the formation of GO aggregates within the GO-loaded CS membrane, leading to the poor interfacial interactions. Such enhancement of TS and EAB was ascribed to the increased CD of the GO-hybridized CS membrane because of the covalent interaction between GO and CS moieties [198]. Wang et al. reported the similar trend in TS of cellulose triacetate-GO membrane suitable for the FO process [199].

#### 4.2.5. Swelling

Swelling study of the membranes can be a potent way to identify and substantiate the stability of novel crosslinks in polymeric membranes, since the SR is inversely proportional to CD. SR deteriorated in the following order: PVA > PVA-silica > PVA-MA-silica membranes, suggesting the increase in crosslinking in PVA-MA-silica membranes [46]. Crosslinking-mediated reduction of hydrophilic character was manifested in the decreased SR of PMDA crosslinked PVA membranes than un-crosslinked PVA. Similar observations were registered for MWCNT-PVA membranes when proportion of MWCNT in PVA matrix was increased from 0.3 to 0.5 wt% [100]. As per Yilman et al., such lowering in SR was ascribed to the enriched hydrophobicity of MWCNT-PVA membranes containing hydrophobic aggregates carrying higher amounts of MWCNTs. Interestingly, with the increase in MWCNT proportion of PVA matrix from 0.1 to 0.3 wt%, substantial increase in SR was observed because of the enhancement in the amorphous region within the polymers via destruction of crystalline structure in-presence of MWCNT, also realized from DSC analyses. Panahian et al. reported similar kind of increasing tendency in SR for multilayer polymeric membranes containing modified-MWCNTs [200].

#### 4.2.6. Hydrophilicity

Enhanced hydrophilicity of PEBA-GNP NCP membranes over unfilled PEBA membranes was realized from the lowering in contact angle for the NCP membrane as compared to the unfilled membrane [121]. Such improved hydrophilicity for increasingly GO-loaded GO-CS hybrid membranes was attributed to the presence of ionic groups (i.e., –COO^−^ and –NH_3_^+^), together with the superficial roughness created by the GO flakes and aggregates [136,175]. Similarly, with the increased addition of Zeolite 3A, elevated hydrophilicity of the composite membrane was observed, resulting in the improved water flux and salt retention [120]. With the increase in thickness of PVA composite membranes, the contact angle was noted to decrease, suggesting continuously enhanced hydrophilicity for the relatively thicker membrane. The hydrophobicity followed the order: PVA < PVA-silica < PVA-MA-silica membranes, as the hydrophilic O–H and –COOH involved largely in the production of lesser hydrophilic ester- and Si–O–Si-based crosslinks [46]. Similar results were obtained when PVA was crosslinked with either GA [80] or PMDA [127], leading to the consumption of some hydrophilic functional groups. Accordingly, with the increased crosslinking, reduced hydrophilicity was observed for PVA composite membranes supported on PVS-based hollow fibers and the PVA-PAN composite membranes [88,122,127]. Indeed, CD was determined by Flory-Rehner equation (Equation (3)):

(3) $$V_{c}=-\left[\ln\left(1-\phi_{p}\right)+\phi_{p}+\chi \phi_{p}^{2}\right]/\left[V_{s}\left(\phi_{p}^{1/3}-0.5\phi_{p}\right)\right]$$

Here, *χ*, *ϕ_p_*, and *V_s_* are the Flory-Huggins solvent interaction parameter (0.494) [201], volume fraction of the swollen polymer, and molar volume of the swelling agent (i.e., water). Initially, the polymer film was immersed in hot water at 80 °C for few hours until a constant weight of swollen polymer film was obtained. Thereafter, from *V_c_*, the average molecular weight between crosslinks (i.e., *M_c_*) was calculated with the help of Equation (4):

(4) $$V_{c}=\frac{d}{M_{c}}$$

Here, *d* is density of polymer. In this context, the density of amorphous PVA (i.e., 1.269 g cm^−3^) was taken into consideration, as the XRD of crosslinked PVA film showed amorphous behavior.

### 4.3. Desalination Performance and Mechanisms

#### 4.3.1. Membrane Structure

Theoretically, permeability and selectivity of membranes during PV are governed by the solubility and diffusivity of components of the membrane. In fact, water permeability of a membrane is governed by the hydrophilicity and availability of free volume within the membrane. It is the difference of diffusion rate between salt and water other than their difference in solubility in the membrane that essentially contributes to the preferential permeation of water in the membranes (Table 3). Indeed, solubility of NaCl in the membranes is very close to that of water, while the diffusivity of NaCl in the membrane is half that of water, which contributes to the high H_2_O/NaCl selectivity of the membranes. Thus, high diffusivity of water over salt is ascribed to the inherent characteristics of dense hydrophilic GO hybridized CS membrane, as the diffusivity of water and salt in membrane is dependent on the property and microstructure of membrane materials [202].

Similarly, all the GNP-loaded membranes demonstrated improved flux and rejection than unfilled PEBA membranes. Such increment in flux should be attributed to the increasing hydrophilicity of nanohybrid membrane during addition of GNPs [121]. Increased flux and hydrophilicity of GNP-filled nanohybrid membranes were attributed to the homogeneously dispersed graphene components acting as water-selective channels [203,204]. In fact, with the increase in GNP content in PEBA from 0 to 3 wt%, total salt rejection was found to enhance from 99.12 to 99.92% at 65 °C. Such improved salt rejection was related to the lengthened tortuous pathway and simultaneously decreased free volume within the GNP-filled PEBA matrix. However, flux was noted to become significantly deteriorated when GNP content was further increased in PEBA, as GNP aggregations affected the passage of water molecules by blocking the pores. In case of GNP-filled polymer membranes, pores are absent in GNP moieties and, hence, GNP is impermeable to molecules or ions. However, if nano-pores can be created inside GNP filled matrix, the matrix can allow the selective passage of the ions. Otherwise, in the absence of nano-pores, GNPs can merely act as fillers, which behave as barriers against the uncontrolled ion passage by extension of the tortuous pathway through the membrane.

**Figure 18:**
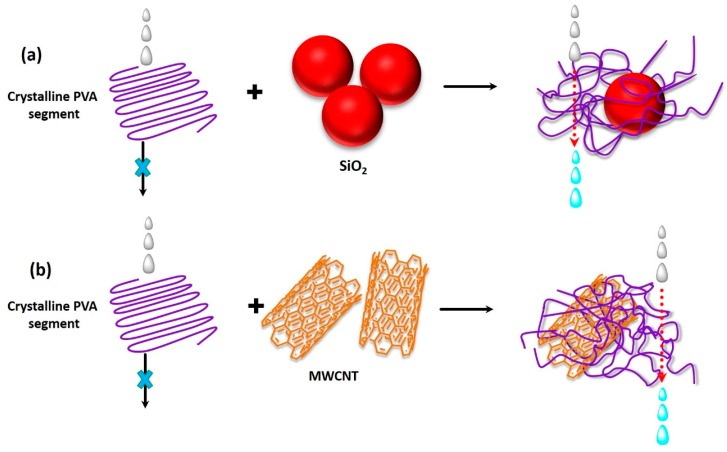
Disruption of polymer chain packing facilitates increased flux in NCP membrane containing (**a**) SiO_2_ and (**b**) MWCNT.

NaX NPs containing PEBA-based hybrid composites demonstrated encouraging rejection of >99%, because of the disruption of polymer chain packing in PEBA and associated lowering in free volume radius [119]. Moreover, enhanced salt rejection was observed in PVA-MA-silica membranes over PVA-silica membranes, as relatively crosslinked and compact nature of PVA-MA-silica membranes prevented the passage of salts [100]. Accordingly, as the silica content was increased further, salt rejection increased because of the consequent increase in the degree of crosslinking. Despite the increase in hydrophobicity of PVA-MA-silica membranes over PVA-silica membrane, water flux increased in PVA-MA-silica membranes as compared to PVA-silica membrane. However, both flux and salt rejection were noted to increase for PVA-MA-silica membranes containing 10% SiO_2_ [46]. Such combined improvement in both flux and salt rejection can be ascribed to the disruption of polymer chain packing by the incorporated silica particles in nano-scale and associated enhancement in free volume. Consequently, diffusion coefficient of the PVA-MA-silica membrane bearing 10 wt% SiO_2_ was noted to be elevated because of the increasingly amorphous segments of PVA-MA-silica membrane facilitated faster diffusion of water (Figure 18a). However, water flux was noted to deteriorate once the SiO_2_ content was increased beyond 10 wt%. Gradually, the elevated hydrophobicity and the compact nature might be the major contributing factors behind such reduced water flux.

A similar kind of observation was also noted for MWCNT-PVA and Zeolite 3A-PEBA composites [120], in which the slight reduction in highly loaded composites could be explained by agglomeration of fillers. For instance, water flux was the maximum for MWCNT-PVA composite bearing 0.3 wt% filler, suggesting the maximum availability of voids because of the creation of such amorphous region that facilitated easier passage of water molecules (Figure 18b) [205,206]. Moreover, water molecules can pass through the hole of MWCNT with less resistance as compared to the PVA matrix [125].

In addition, similar to PVA-MA-silica membrane containing intermediate level of SiO_2_ (i.e., 10 wt%), MWCNT-PVA composite containing 0.3 wt% filler showed better salt rejection than the composites containing 0.1 and 0.5 wt% MWCNTs. In fact, higher aggregation in MWCNT-PVA composite possessing 0.5 wt% filler was the major reason behind such inferior salt rejection. Indeed, deteriorated flux was observed for the PVA composite membrane prepared with the increasing amount of MA [114] or PMDA [127] as crosslinker, which was possibly because of the reduced hydrophilicity as a result of consumption of O–H of PVA, along with the relatively compact structure and thus, lesser availability of free volume in the crosslinked structure. Indeed, such a crosslinked structure might contribute significantly in the enhanced salt rejection up to 99.9% for the feed saline of 50,000 ppm [114].

In case of PMDA crosslinked PAN-PVA composite membranes, crosslinking should be desired at the optimum level to ensure the minimum availability of –COOH, which in-turn influence the facilitation of the water transport through the membrane [127]. In this context, the highest performance for PAN-PVA composite membrane could be achieved when crosslinking was conducted at 100 °C. However, beyond 100 °C, water flux deteriorated excessively whereas salt rejection performance was found to be <99% when crosslinking was conducted below 100 °C, since crosslinking could not be completed below 100 °C.

**Figure 19:**
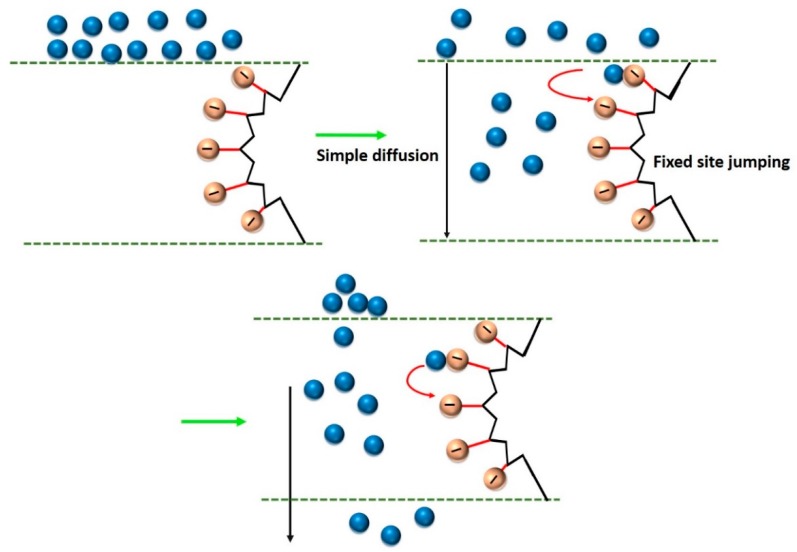
Mass transport of water molecules through an SPTA crosslinked PVA layer by simple diffusion and fixed site jumping.

For SPTA crosslinked PAN-PVA composite membranes, –SO_3_H contributed significantly in maintaining water flux, despite increased crosslinking [128]. Clearly, a higher content of SPTA crosslinker is beneficial to water transport. Therefore, permeation of water through S-PVA layer was a combination of two mechanisms, of which the first mechanism is based on solution-diffusion, wherein water dissolves in the upper surface of SPVA layer, diffuses through the free volumes in the S-PVA polymer, and desorbs to the downstream atmosphere (Figure 19). The second mechanism is the –SO_3_H-assisted transportation, by which water molecules are adsorbed to –SO_3_H of the membrane surface, followed by jumping to the nearby –SO_3_H of the bulk, until reaching the other side of the S-PVA layer, and finally desorption at the permeate side [207,208]. In fact, higher SPTA content in S-PVA films enhances the interaction probability of water molecules with –SO_3_H, suggesting rapid binding of more carriers with the permeate on the membrane feed side. Such passage of permeate reduced the mass transport resistance in S-PVA films, transforming the convex water concentration profiles into the rectilinear shapes in the permeate direction [209].

In the case of the Zeolite 3A-PEBA membrane, the cage size is an important parameter for allowing higher rejection value. Thus, excellent salt rejections of >99.67 were achieved for all the Zeolite 3A-PEBA membranes, as most of the contaminating ions have larger molecular diameter than the cage size of zeolite or restricted free volume of the polymer [81,120]. Moreover, active deposition of crystalline particulate matter of inorganic salts and organic foulants, such as humic acid, can actuate the time dependent deterioration of flux through PV membrane, as the deposited layer can block the passage of water via occupying hydrated salts in the free volume of PVA network [164].

In this context, because of larger molecular size, organic foulants possess lesser tendency to penetrate into the bulk of membrane. However, they reside at the surface as a layer susceptible to microbial degradation. Moreover, it was noticed that foulants might combine with Ca^2+^ to produce insoluble Ca salts, which efficiently prevents water transport because of blocking the pores. Membranes equipped with anti-foulant properties can easily be cleaned by water solution and, thus, the fouling layer was strategically not allowed to penetrate deeply or associated with the membrane with stronger bonding force. It is well known that the PV performance not only depends on the membrane properties, but also on the operational conditions, such as feed concentration, temperature, permeate pressure, and feed flow rate [210].

#### 4.3.2. Feed Concentration

**Figure 20:**
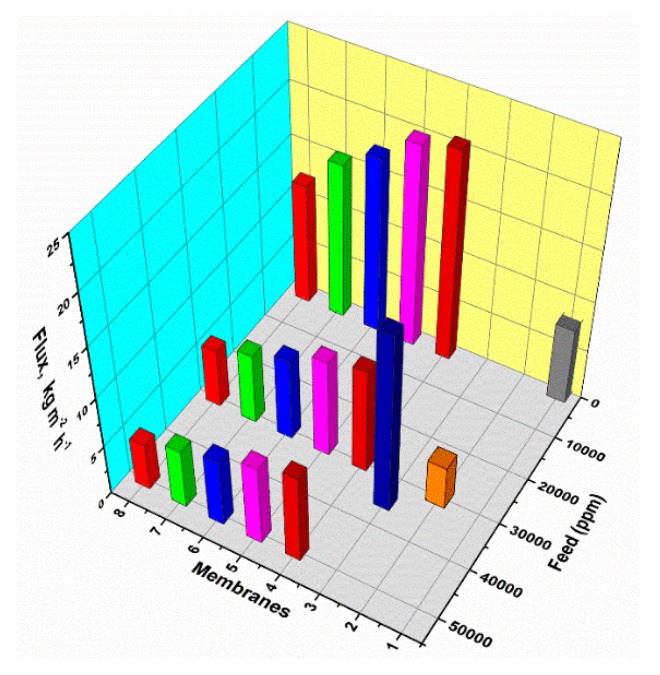
Variation of flux as the function of feed concentrations of different membranes (1: PEBA/PAN/PE, 2: 20zeolite 3A/PEBA, 3: GO/CS, 4: PVA-SiO_2_/PVSF hollow fiber (thickness: 0.22 ± 0.03 μm), 5: PVA-SiO_2_/PVSF hollow fiber (thickness: 0.30 ± 0.03 μm), 6: PVA-SiO_2_/PVSF hollow fiber (thickness: 0.60 ± 0.05 μm), 7: PVA-SiO_2_/PVSF hollow fiber (thickness: 0.85 ± 0.07 μm), and 8: PVA-SiO_2_/PVSF hollow fiber (thickness: 1.13 ± 0.11 μm) at 60 °C.

The feed concentration is believed to affect directly the sorption of its components at the interface between feed and membrane surface [210,211,212]. With the increase in salt concentration, reduction in the vapor pressure of water occurs, accompanied by the slight reduction in membrane swelling. Therefore, permeate flux is reduced (Figure 20) [213]. In general, the effect of salt concentration on water flux is dependent on feed temperature. In fact, salt concentration has a negligible influence on water flux at room temperature [100,119]. However, with the increase in feed temperature, water flux was noted to decrease with the increase in salt concentration [50,100,126,127,136]. Such an adverse effect of increased NaCl concentration on the reduced water flux was observed for pervaporative desalination by the MWCNT-PVA membrane at 40 °C [125] and the PEBA-Zeolite 3A membranes [120]. The gradual reduction in the water of feed solution with higher NaCl concentration affect PV significantly, since the driving force for the pervaporative separation is primarily the difference in the partial pressures of the components on the two sides of the membrane. Importantly, vapor pressure is exponentially related to the temperature and, thus, lowering of the water concentration imposes a pronounced effect on the concentration of water at the membrane surface that ultimately reduces the difference between the partial pressures of the water on either sides of the membrane, hampering the diffusivity and flux at the higher temperature. However, a substantial effect of increased salt concentration of the feed solution in reducing the water flux was observed for PV-based separation by the GO-PAN membrane even at 30 °C [50]. With the increase in feed concentration in the pseudo-liquid type aqueous salt solution, increased relative population of bulky hydrated ions and accordingly decreased concentration of free water molecules at the adjacent region of membrane surface was responsible for the lowering of water flux through membrane. Similar flux declines with the increased feed concentration were reported in seawater desalination through PV [113,214] and MD [215,216,217] processes. Indeed, the decrease in flux for more concentrated feed can be explained by the reduction in water VP. Such reduction in VP of saline water can be interpreted from Equation (5) showing the relation between VP of saline water (*P*) and pure water (*P*_0_):

(5) $$\log\left(\frac{P}{P_{0}}\right)=hS+jS^{2}$$

Here, *S* and *h*/*j* represent salinity (i.e., the amount of salt in water) and constants, respectively.

#### 4.3.3. Feed Flow Rate

Xie et al. evaluated the impact of feed flow rate on water flux, while keeping all the other parameters unaltered [100]. According to their observation, the feed flow rate showed little or negligible effect on water flux through hybrid PVA-MA-silica membrane. In this context, the feed flow rate was varied while maintaining the Reynolds numbers within 21–105 to ensure laminar flow of the feeding mixture. In general, an increase in feed flow rate reduces concentration polarization and increases flux because of the reduction of transport resistance in liquid boundary layer [210]. On the contrary, such an observation made by Xie et al. indicated that mass transfer from the feed to the membrane was not a rate-limiting step and, thus, the rate of PV was not affected by the feed flow rate. However, it was not reported that what will happen to the rate of PV if the feed flow rate is increased to the turbulent flow region so that the Reynolds number exceeds beyond 4100.

#### 4.3.4. Effect of Permeate Pressure

Theoretically, the maximum flux is achieved when the permeate pressure reaches absolute zero. In doing so, a reasonable cost factor is involved for maintaining high vacuum. In general, the water flux decreases when the permeate pressure is increased because of the consequent decrease in the driving force, necessary for mass transport [119]. As per the observation made by Xie et al. [100], once the permeate pressure exceeded 15 Torr, the driving force for water vaporization approached near zero, leading to the negligible net evaporation and, consequently, to the low mass transport of water. Such a phenomenon is closely related to the fact that the saturation VP of water is about 2.27 kPa. Thus, as the permeate pressure was increased beyond 2 kPa, almost a 90% drop in the diffusion coefficient was observed, indicating a tremendous reduction in the diffusion of water through the membrane [100].

#### 4.3.5. Temperature

Increasing flux with temperature is the prevalent observation for PV separation studies [218,219]. As VP is exponentially related to temperature [100,220], increased temperature up to a certain level resulted in the enhanced flux, which is again because of the increased driving force between the sides of the membrane created by the increasing VP in the feed side [119,121,126]. Such an observation can be interpreted by the Antoine equation (Equation (6)), which expresses an exponential relation between VP (i.e., *P*) and temperature (i.e., *T*) of water [221]:

(6) $$\log\frac{P}{P_{0}}=A-\frac{B}{T+C}$$

Here, *A*, *B*, and *C* are the constants.

Furthermore, with the increase in operational temperature, exacerbated thermal motion of water molecules in solutions can accelerate the diffusion of water through the membrane. In addition, the mobility of the polymer chains in a membrane increased with the feed temperature, leading to the increased free volume in the membranes. According to the free volume theory [222], such a temperature-dependent increase in free-volume can be ascribed to the enhanced frequency and amplitude of the polymer chains in amorphous regions, creating momentary free volumes in excess at higher temperatures [119,223]. Consequently, greater free space is available for water molecules to diffuse easily through the membrane. Thus, with increasing temperature, flux was noted to increase exponentially for all the membranes at all the feed concentrations [114,118]. However, such enhanced free volume at higher temperatures is responsible for the deterioration in the salt rejection capability, as enhanced thermal motion of polymer chain segments allowed salt-ions to pass through the membrane [118].

#### 4.3.6. Thickness of Membrane

With the increase in membrane thickness, resistance across membrane increases [46,218,224,225]. Accordingly, water flux was found to decrease from 5.12 to 2.73 kg m^−2^ h^−1^ when the PEBA-NCP membrane thickness was altered from 72 to 181 μm [121]. A similar decrease in flux with the enhancement in membrane thickness was observed by Xie et al. [46]. However, the thickness variation did not remarkably affect the extent of salt rejections.

In this context, water permeabilities through S-PVA-PAN composite membranes were found to reduce drastically with the increase in thickness of S-PVA layer from 76 to 1.06 μm [128] because of the significantly lower water diffusivity at the dry portions of the membrane, closer to the permeate side. Interestingly, in dry regions, water flux is the function of diffusivity, which was reduced slightly because of the higher membrane thickness [135,209,226].

According to Liang et al. [50], thicker deposition of GO increased the thickness of GO-PAN composite membrane, which affected water flux and membrane efficiency [227]. Therefore, in order to fabricate high-efficiency PV membranes, the minimum thickness of the GO layer should be preferable, along with the maintenance of the required structural integrity. It appeared that the increased thickness of the GO layer should compel the diffusing water molecules to travel through a substantially longer tortuous pathway, constituting of the nano-capillaries between well-stacked GO sheets [228,229,230], resulting in the significant lowering of mass transport through the relatively thicker GO-PAN composite membrane.

A similar influence of increasing thickness was observed in the case of composite PVA membranes [88]. In fact, composite membranes based on dilute PVA solution-coated PVS hollow fiber exhibited higher flux (up to 7.4 L m^−2^ h^−1^) as compared to those membranes prepared using less dilute-solution-coating, relatively concentrated PVA solution produced the membranes of higher thickness offering higher resistance against the mass transport [227].

On contrary, the increased flux was resulted using thin membrane obtained from less dilute solution coating [118]. In the case of composite membranes, the non-linear variation between membrane thickness and flux was ascribed to the cumulative effect shown by layer thicknesses of top and support layers. These two layers were composed of different materials carrying different porosities and structures. Moreover, steep decline of flux was resulted in the case of increasingly dense support structure, along with the increase in the top membrane layer thickness [231].

#### 4.3.7. Electrical Resistance of Membranes

**Figure 21:**
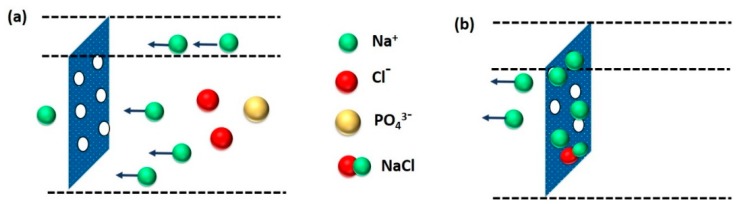
Two-stage separation of membrane comprising (**a**) size exclusion and (**b**) both size and charge exclusion.

It is well established that the PV mechanism is mostly interpreted by the solution-diffusion model, constituting three steps: firstly adsorption and dissolution of feed molecules on the membrane surface, followed by diffusion of dissolved species through the membrane, and finally, desorption of the diffused species at the permeate side [50]. In addition to the usual solution-diffusion model, water passage or salt rejection in the PV desalination system is directly related to the size exclusion and/or charge exclusion phenomena (Figure 21) [81,218]. Electrical resistance of the membrane is an important parameter, which helps evaluate the charge characteristics of the membrane involved in separating charged species in PV desalination system. Thus, PV desalination can be subdivided into two major periods, of which in the first period, water passage and diffusion through the uncharged membrane is related to the size exclusion process, because the size of the molecules is the sole deciding factor to separate the unwanted species from water. Later, during the subsequent period, once the surface of the membrane becomes charged by the ions in seawater, an electrostatic interaction starts to prevail between the membrane surface and seawater. Consequently, water passage and diffusion through the membrane is jointly controlled by both charge exclusion and size exclusion mechanisms (Figure 21). Accordingly, when an uncharged membrane becomes charged during the last period of PV desalination, the electrical resistance of the membrane should be altered.

In the case of the PEBA-GNP composite membrane, the electrical resistance of PEBA membrane was noted to decrease after desalination. Accordingly, the conductivity and the charge load on membrane increased during desalination, as the hydrated ions penetrate and reside in the feed side of the membrane structure. Thus, this increasingly charged membrane repels the existing ions in the feed side of the membrane, and hence, the incoming ions experience repulsive force towards the feed side according to the ‘charge exclusion’ theory. Moreover, during addition of GNP moieties, the initial electrical conductivity of the uncharged membrane increased, if GNP moieties are devoid of forming agglomerates inside the membrane [121]. Such increased conductivity helps improve the performance of PV desalination via enhancing the charge exclusion ability of the membrane, in addition to the enhanced size exclusion ability through extending the tortuous pathway through the GNP-loaded membrane.

For the PVA-coated composite membrane, the purity of the produced water was evaluated by measuring the conductivity [114]. Chaudhri et al. observed the lowest conductance of ~20 μS cm^−1^ when a membrane of 1000 nm top-layer-thickness was used to purify feed water containing 30,000 ppm NaCl. However, with the reduction of thickness, the conductance value of the produced water was noted to increase because of diffusion and percolation of some solvated NaCl through the swollen membranes.

## 5. Inorganic Membranes for Pervaporative Desalination

Recently, polymeric membranes have found extensive applications for membrane-based desalination and wastewater treatment because of sustainability, selectivity, and high performance potential. Use of polymeric membranes in desalination and wastewater treatment depends on the optimization of the two opposite terms, i.e., permeability and selectivity, along with membrane fouling and scaling. Poor sustainability restricts the use of the polymeric membranes as water purifier and thus, substantial attention was introduced for the development of thermomechanically stable and sustainable MMMs and thin film NCPs [232]. In fact, composite membranes comprising of the blended properties of both polymeric matrix and inorganic fillers are gaining high insight for membrane-based desalination techniques.

As compared to the organic membranes, inorganic membranes usually possess superior structural stability, i.e., no swelling and compaction, even in harsh chemical environments and high temperatures [233,234,235]. In addition, desalination of wastewater containing radioactive substances and highly concentrated organic effluents, oil, and grease can effectively be carried out using inorganic membranes instead of the polymeric membranes, which suffer from severe fouling and poor sustainability [236,237,238,239]. Additionally, inorganic membranes are lesser susceptible towards bacterial attack as compared to the most of the polymeric membranes [240]. Moreover, inorganic membranes are beneficial for brackish water treatment, seawater pre-treatment, and high temperature desalination, wherein the high rejection (i.e., >99%) is not an essential task [241]. Therefore, development of inorganic membranes possessing extraordinary physicochemical properties, high tunability, and recyclability/reusability are gaining high insight. The selective transportation of constituents through inorganic membranes are principally guided by the pore size of the membranes. In this context, inorganic membranes can broadly be classified into two categories, such as ceramic- and carbon-based membranes. Oxides, such as alumina, silica, titania, and mixtures of these components are the most commonly used ceramic membranes [242]. Of these, amorphous silica contains extremely small pores and an asymmetric structure, wherein the microporous silica prevails onto a support containing several α- and γ-alumina layers [243,244]. Most of the reported silica membranes possess either flat plate or tubular geometry. The flat plate geometry usually possess small surface area of ~10^−2^ m^2^ because of the limitations of dip-coating technique. However, because of larger surface area, tubular-shaped silica membranes are better suitable for pervaporative desalination [235]. Instead of such advantages, the tubular-shaped membranes possess certain disadvantages, such as expensive and low surface area-to-volume ratio (i.e., <500 m^2^ m^−3^).

Ceramic membranes, another extensively used inorganic membrane, is composed of a macroporous support layer and a meso-/micro-porous active layer. Initially, high cost of synthesis and complex instrumentation limited the industrial use of inorganic membranes for pervaporative desalination [245]. In past days, inorganic membranes were merely used to work under highly contaminated feed environment and at high temperature, wherein the polymeric membranes did not work properly. However, the discovery of low-cost ceramic membranes containing clays and organic pore formers has reinvigorated the frequent use of ceramic-based inorganic membranes in the commercial scale [246,247,248,249]. In fact, in the modern era, syntheses of new inorganic-based composite membranes, such as MOF, CNT, and graphene and its derivatives, have gained a high impact for the pervaporative desalination of wastewater. These membranes have imparted the high permeability, selectivity, and salt rejection. Therefore, these new membranes are anticipated to ameliorate some existing issues found in the contemporarily used materials and accomplish the practically efficient, high productivity, and energy saving wastewater treatment processes.

### 5.1. Various Inorganic Membranes Used

Korngold et al. optimized the effects of membrane properties (i.e., thickness and charge density) and experimental conditions (i.e., temperature, feed water salinity, and sweep gas velocity) on water flux during pervaporative desalination of a nonporous proton exchange membrane [42]. Interestingly, the water flux was found to increase with the rise in velocity of sweeping gas to attain a plateau at the sweeping gas velocity of 2 m s^−1^, attributed to the hydraulic resistance, which was imposed by the hollow fiber membrane. Alternatively, the passage of water through the membrane depends on the transport of the liquid within the membrane and is independent of air velocity. The permeate flux increased with the decrease in membrane thickness and the increase in water temperature. However, permeate flux reduced with the enhancement of feed NaCl concentration from 0 to 3 M. In most of the cases, the salt rejection efficiencies through different membranes in pervaporative desalination have been reported to be ~99%. However, because of the permeation of both solutes and water through hydrophilic inorganic membranes, desalination efficiency reduces substantially [43,137,250]. The performance of various inorganic membranes are discussed in Table 4.

The permeation of salts through hydrophilic nonporous membranes occurs through different possible mechanisms [251,252,253], of which the most common mechanism includes the adsorption of salt molecules into membranes, followed by smooth passage through the polymer matrix by diffusion. In fact, the salt permeation was found to be significantly affected by several important membrane-based factors, such as the temperature, the free-volume space within the membrane, the hydrodynamic radius of the dissolved substances, the closeness of solubility parameters between membrane and component, and the possible electrostatic interactions between membrane and substance. According to Ju et al. [254], water selectivity of the membrane decreases with the increase in permeability.

#### 5.1.1. Ceramic Membranes

**Figure 22:**
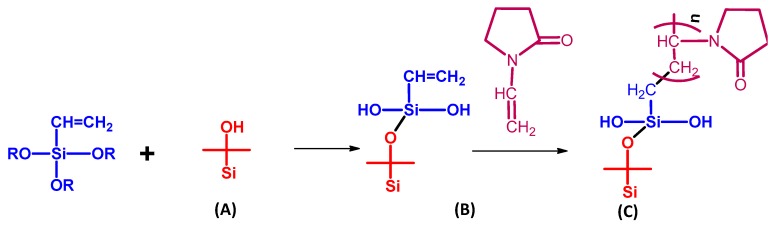
Schematic diagram of the surface modification procedure: (**A**) Surface silylation, (**B**) graft polymerization, and (**C**) resultant covalently-bonded PVP chain.

**Figure 23:**
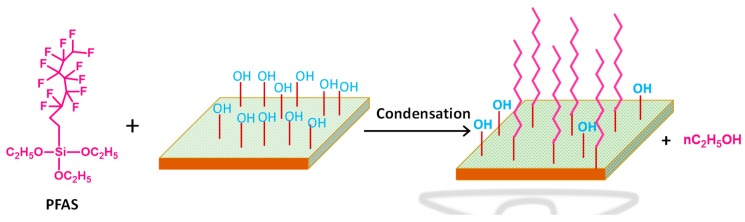
Scheme of modification process by PFAS molecules.

Recently, the use of ceramic membranes in UF, NF, RO, GS, and PV is gaining high insight [258,259,260,261]. The foremost application fields of ceramic membranes include food, biotechnology, water purification, and pharmaceutical industries. In pervaporative desalination, use of ceramic membranes possess several advantages over polymeric membranes, such as thermo-mechanical resistance, chemical stability, sustainability, non-swelling, and simple cleaning. Commercially viable ceramic membranes are synthesized from metal oxides, such as alumina, silica, zirconia, and titania. Because of the prevalence of O–H groups, such hydrophilic ceramic membranes can easily bind with water molecules [258,259,260,261]. Therefore, the incorporation of hydrophobicity into the predominantly hydrophilic ceramic membranes via grafting of hydrophobic molecules and chemical modifications is recently gaining high insight [262,263,264,265,266,267,268,269,270]. Alami-Younssi et al. [262] and Jou et al. [263], grafted γ-alumina and polyvinyl acetate into ceramics for chemical modification. Again, Pickard et al. [264], investigated the impregnation of two fluorinated silanes, C_6_F_13_C_2_H_4_Si(OMe)_4_ and C_8_F_17_C_2_H_4_Si(OEt)_3_ into the ceramic membranes. Additionally, Caro et al. [265], reported the chemical treatment of commercial ceramic filters to achieve both pore narrowing and hydrophilic/hydrophobic functionalization. The γ-Al_2_O_3_-based top layer of the asymmetric ceramic filter was functionalized by in situ hydrolysis of tetraethylorthosilicate to introduce Si–OH-rich hydrophilic nanoporous SiO_x_ top layer, followed by silylation of the γ-Al_2_O_3_ layer, to attain organophilic functionalization (Figure 22), and finally, reaction of the γ-Al_2_O_3_ layer with alkyl/aryl phosphonic acids to incorporate hydrophobic behavior. In this context, the use of FAS for the introduction of hydrophobic character via reaction between O–H of the ceramics and ethoxy groups (O–Et) of organosilane compounds [262,264,270,271,272]. The as-prepared hydrophobic layer of organosilane compounds onto ceramic surface caused the hydrophobic character of such membranes [264,268,273] (Figure 23).

In this context, Larbot et al. reported the synthesis and applications of hydrophobic ceramic membranes via grafting of different FAS onto the surface of alumina, titania, zirconia, and silica membranes [264,270,274,275,276].

#### 5.1.2. Metal Oxide Membranes

Ceramic membranes are composed of a wide range of insoluble oxides, of which zirconia- and titania-based mesoporous membranes find extensive commercial applications. From the very beginning, porous metal oxide-based ceramic membrane is composed of the multi-layer asymmetric structure containing a thicker supportive layer of comparatively larger pores (~500 nm) for attainment of the mechanical integrity, an intermediate layer, which reduces pore size into mesoporous dimensions (2–5 nm), and a thinner top layer possessing small pores (<1 nm) responsible for selective separation [277,278]. Metal-oxide based ceramic membranes are predominantly synthesized via sol-gel method, which can further be classified into two categories, i.e., colloidal and polymeric sol-gel routes, depending on the solvent used. In colloidal sol-gel method, water is used as solvent, whereas in polymeric method, organic solvents are utilized. Therefore, colloidal method is more environment friendly than polymeric sol-gel method [279]. Synthesis of ceramic membranes from oxide precursors via colloidal sol-gel method comprises of two steps, of which the first step is associated with the precipitation of a condensed, hydroxylated species from hydrolyzed precursors. However, in the second step, the as-prepared precipitate is converted into a stable sol via a peptization reaction with basic or acidic electrolytes [280].

Mesoporous γ-alumina membrane, one of the most widely investigated ceramic membranes, is usually considered as the interlayers for ceramic membranes. Though most of the mesoporous γ-alumina membranes contain the average pore size within 3–5 nm [280], membranes showing average pore sizes of ~1 nm has also been studied. However, such low pore dimensions restrict the permeation of water through membranes resulting in very low permeance, which is not suitable at all for industrial applications. However, such a problem was surmounted by Wang et al., via synthesizing solvent resistant alumina NF membrane of 1.61 nm thickness, possessing the permeability as high as 17.4 L m^−2^ h^−1^ bar^−1^ [281]. Though the orthodox metal oxide-based ceramic membranes possess three layers, the newly developed ceramic membranes contain mesoporous top layer for pervaporative desalination [278,282,283,284,285,286]. These membranes contain several advantages, such as low cost-synthetic technique, since these membranes are devoid of an extra top layer and involvement of cheap reactants, and reduced membrane thickness, which can enhance the water flux via minimizing the resistance against water diffusion through the membrane. In this context, coal fly ash, the by-product of combustion of raw coal in power plants, was used as raw materials for fabricating mullite- (3Al_2_O_3_·2SiO_2_) and cordierite-(2MgO·2Al_2_O_3_·5SiO_2_)-based ceramic membrane supports, along with the addition of dolomite for controlling porosity, pore size distribution, and microstructures [287,288,289].

Selective desalination through the majority of porous metal oxide-based ceramic membranes is fabricated via increment in size by hydration, followed by electrostatic effects at the pores [290]. For decontamination of raw industrial wastewater bearing the mixtures of toxic metal ions, organics, and inorganic substances, the selectivity is primarily achieved via electrostatic interactions between pollutants and positively-charged metal oxide ceramic membranes [291]. Electrostatic interaction, pH of the feed solution, and modulation of the membrane surface charge influence the rejection mechanism.

**Figure 24:**
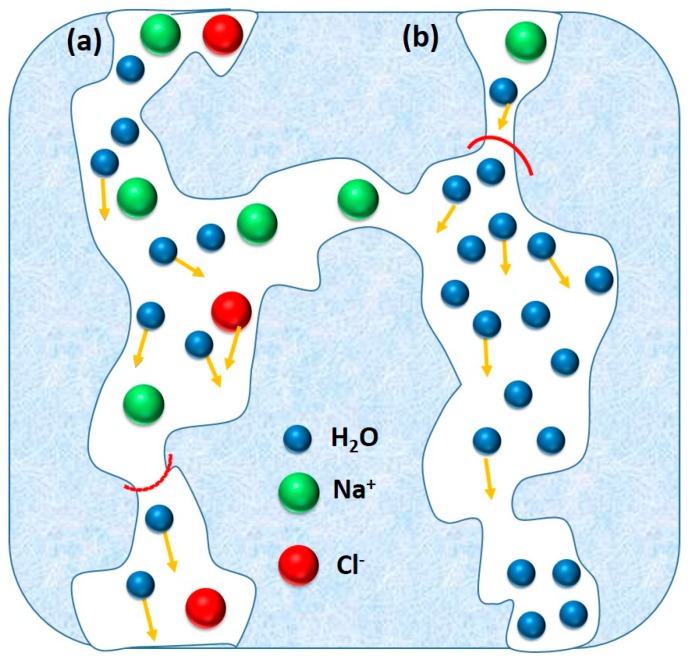
Illustration of percolative porous pathway and diffusion of water and hydrated salt ions through (**a**) mesoporous region that non-selective to small amount of hydrated salt ions, and (**b**) microporous constrictions that only allows the passage of water molecules.

Though the maximum ceramic membranes possess uniform meso-/micro-porous structure, some oxide-based ceramic membranes, especially amorphous silica membranes exhibit trimodal pore size distribution, containing smaller (i.e., 3 Å) and larger (i.e., 8 and 12 Å) pores, responsible for selective permeation [282]. Desalination is primarily achieved by the restriction of hydrated ions through pores, allowing the passage of water molecules (Figure 24a). However, selective permeation through mesoporous portions connected with the microporous shrinking or to another mesoporous region may allow the passage of small amount of salts in the permeate side (Figure 24b), which would reduce the selectivity towards desalination.

Additionally, removal efficiency of membranes can be enhanced via combination of filtration and adsorptive phenomenon of the reactive surface functional groups, such as the active silanol group present onto the surface of nanosilica membrane, can impart adsorptive active sites to adsorb pollutants from aqueous solution [292]. Since superficial properties play the pivotal role to affect several membrane-based intrinsic properties, such as hydrophobicity, surface charges, hydrodynamics, and fouling rate [293], extensive investigations have been devoted for the proper characterization of superficial properties of the ceramic membranes.

The surfaces of the metal oxide-based ceramic membranes usually possess significant numbers of O–H enhancing the hydrophilicity of membranes [294]. These O–H located on membrane surface provide the necessary reactive sites to graft hydrophobic organic moieties, necessary for the introduction of hydrophobicity into the predominant hydrophilic membrane. Such hydrophobized ceramic membranes find widespread applications in membrane-based operations, such as desalination, distillation, and PV, as compared to the native hydrophilic metal oxide membranes [295].

The hydrophobicity and/or shrinking pore size of ceramic membranes carrying chemically active surface functional groups, such as silane groups, can be controlled by grafting FAS to the metal oxide and/or membrane surfaces [296,297,298,299]. As stated earlier, the dual functionalities of FAS, i.e., hydrolyzable groups in one end and organic moieties on the other, can easily react with O–H located onto metal oxide surface to form stable Al–O–Si type covalent bonds. Kujawa and coworkers reported the modification of tubular and planar ceramic titania membranes and powders of Al_2_O_3_, TiO_2_, and ZrO_2_ using varied FAS molecules to obtain hydrophobic membranes of enhanced thermal stabilities [296,297,298,299]. Alternatively, in the presence of water, the reaction between hydrolyzable groups of the organosilanes and other organosilanes for producing a polymeric layer can be another plausible binding route for hydrophobization.

Few researchers have reported the coating of a thin polymeric layer onto commercialized macroporous ceramic membranes for improving the desalination performance of the ceramic membranes [300,301,302,303,304,305,306,307]. The polymeric chains are covalently bonded with the ceramic support surface in such composite membranes, wherein the desired flux and selectivity are achieved from the thin coating and the ceramic support renders the structural fragility and reduces the pore size of the ceramic support [278,308,309]. Since the polymer solution may penetrate into the ceramic pores, preparation of a dense and defect-free composite membrane is highly challenging. Moreover, it is highly essential to rationalize the mechanism of polymer layer formation at the adhesion surface to optimize the desalinating performance, since selectivity and permeation through polymeric membranes are predominantly dependent on the composition, synthetic method, thickness, and operating conditions [310]. In fact, such surface modification modulates permeability characteristics and enhances flux in response to temperature [311,312,313,314,315], pH and controlled phase inversion parameter determining pore structures [316,317,318,319], and ionic strength of the feed stream [320,321,322,323,324]. Polymer coating onto ceramic membranes is carried out mainly via dip-coating method, in which a coating solution containing polymer, solvent, and crosslinker is prepared, followed by coating onto the outer surface of the tubular ceramic support and heat treatment. In order to enhance the surface smoothness and ensure supreme quality of the resultant polymer-ceramic composite membranes, the ceramic supports are thoroughly polished prior to the dip-coating [301]. Additionally, tubular α-alumina ceramic membrane has also been coated using CA to reduce the original ceramic pore size in order to increase the efficiency of the composite membranes [300]. The coated polymer layer also plays an important role in controlling the pore-type diffusion and sorption of components onto the coated surface to attaining the better removal efficiency.

#### 5.1.3. Zeolite Membranes

Zeolites and crystalline aluminosilicates carrying well-defined pore/channel structures are gaining high insight for alternative inorganic membrane-based gas [325] and liquid phase separations [326] and pervaporative desalination [306,327,328,329,330,331,332,333,334,335,336,337,338]. The prevalence of several mono-/divalent cations, such as Na^+^, K^+^, Ca^2+^, and Mg^2+^, enable the cation-exchange phenomenon during desalination. The main advantages of zeolite membranes are directly related to their high separation performance, catalytic activity, good thermal and chemical stabilities, and low fouling tendencies [339,340]. Such membranes are believed to provide desired separation potential for molecules comprising of the variable sizes and adsorptive properties [242].

Zeolite membranes are more advantageous over the conventional polymeric membranes, since the pore sizes of zeolite membranes can be tuned precisely to achieve perfect molecular size and shape for obtaining the high separation [341]. Zeolite membranes are advantageous for desalination because of their ability to remove ions from aqueous saline solutions, leading to high salt rejection [334]. Most importantly, separation efficiency has been found to be directly proportional to the valency ion, indicating that the separation mechanism is based on rejection of the hydrated ions by size exclusion and ions interactions, which are influenced by the charged double layer formed in inter-crystalline gaps of zeolite membranes [139]. If salt rejection cannot be obtained via pore size exclusion, functionalization of zeolite cage can be carried out alternatively to enhance the membrane separation efficiency, as observed in Al-rich zeolite membranes [342], wherein the membrane properties, such as surface hydrophobicity and charge are significantly changed [343].

The mechanism of selective diffusion of water from the salt solution is based on the differences of molecular sizes of water and hydrated ions. The molecular dimensions of water, Na^+^, and Cl^−^ are 2.6, 7.2, and 6.6 Å, respectively [139,142]. Therefore, zeolite membranes of pore sizes within 2.7–6.5 Å should be ideal for salt rejection.

Of different zeolite membranes NaA and MFI membranes are found to possess pore sizes of ~4 Å [344] and ~5.5 Å [345], respectively. Thus these membranes are perfectly suitable for seawater desalination. MFI-type zeolite possesses orthorhombic crystal symmetry with almost cylindrical 10-member ring channels with aperture size within 0.54–0.56 nm and intracrystalline pores within 1.1–1.2 nm [346,347,348]. Indeed, flat, tubular mono, and multi-channel MFI membranes with varied sizes have been fabricated for pervaporative desalination. In addition to the production of high-quality reusable water, MFI-type membranes possess high tolerance level towards long-term strong chlorine cleaning.

Additionally, hydrophilic zeolites, such as LTA membranes, have been found to possess good ion rejection efficiency, yet poor water flux for seawater desalination, because of small pore opening of ~0.4 nm [349], restricts their use for desalination. Additionally, some other classes of zeolite membranes, such as LTA and FAU [350,351], have also been fabricated for membrane-based water purification and desalination.

**Figure 25:**
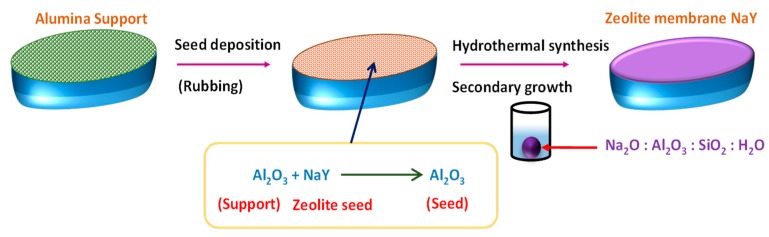
One-step method for the preparation of the zeolite membrane.

**Figure 26:**
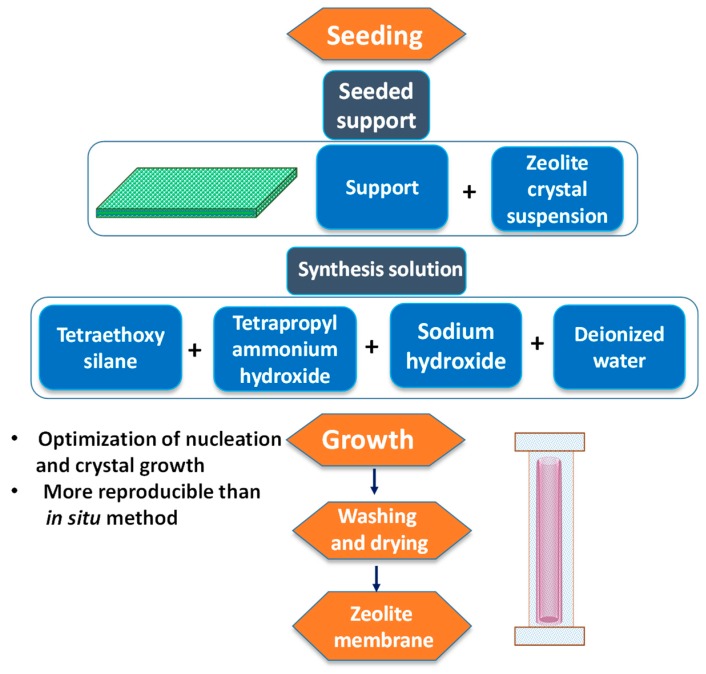
Secondary growth for preparation of the zeolite membrane.

In recent past, zeolite membranes possessing variable morphologies, compositions, and separation prospects have been synthesized. However, the synthesis of zeolite membranes can be carried out either in one step (Figure 25) or via secondary growth approach (Figure 26) [242]. Zeolite nanocrystals, better known as nanozeolites, can readily be used as nanoseeds to fabricate dense zeolite films and membranes via secondary growth based on the superior colloidal properties of their suspensions [352,353]. During secondary growth method, coating of zeolite seeds on the support surface is carried out prior to hydrothermal synthesis to fabricate high quality zeolite membranes. Such method of synthesis gives the better control on membrane formation because of the separation of crystal nucleation and growth within a small crystallization time [354,355,356]. Additionally, the secondary growth method enables the growth of zeolite crystals onto the support rather than in the solution that confines the nucleation transformation to other crystals [357,358]. However, two major challenges appear during membrane synthesis via secondary growth method. The first one is how the colloidal suspension of zeolite particles are prepared, whereas the second concern is how the coating of seeds are carried out onto the support surface.

Coating of seeds onto the support surface may be carried via dip coating [359,360,361,362], rub coating [363], spray coating [364], spin coating [365], and pulsed laser deposition [366,367]. Of these, dip coating is the most widely used method. However, the close attachment of zeolite seeds with the support surface is not possible and, hence, the colloidal suspensions can easily drizzle during removal of supports from suspension. Thus, it is highly difficult to obtain uniform and continuous seeding layer. Moreover, the same coating process is to be repeated several times for obtaining seeding layer with good coverage [368]. Furthermore, the fragility of the substrate and poor compatibility between the zeolite thin film and substrate has imposed the probability of defect formation, which hampers the practical application in commercial desalination. Therefore, fabrication of natural, dense zeolite membranes, devoid of macroporosity or fragile crystal grain boundaries, is gaining high insight.

Dense clinoptilolite has been chosen as one of the promising geomorphic natural zeolites, which shows the desired mechanical robustness in industrial applications. Microwave-assisted heating based on secondary growth has also been attempted to synthesize LTA, MFI, AFI, FAU, SOD, and ETS-4 type zeolite membranes. Such microwave irradiation is more profitable as the method is kinetically fast, in which the resulting membrane is devoid of intracrystalline defect with high salt rejection efficiency [369]. The overall membrane thickness can be controlled in the microwave irradiation method which facilitates the permeation of water at the zeolite film-substrate interface, enhancing the water flux [57].

Though the use of zeolite membranes was initially limited towards GS and ethanol-water separation through PV [346], presently the use of zeolite membranes for desalination is gaining high insight [349]. Historically, Li et al. first reported desalination using MFI silicalite-1 zeolite membrane that exhibited ~77% salt rejection and very low water flux [329]. Therefore, subsequent modifications have been carried out to modify the zeolite structure to improve the salt rejection efficiency, since desalination through zeolite membranes is devoid of the costly pretreatment for polymeric RO membranes. To improve hydrophilicity, the Si:Al ratio of zeolite membranes should be kept as low as possible. In fact, at higher Si:Al ratio, the thermo-chemical stability of zeolite membranes are not satisfactory because of the higher stability of Si–O as compared to Al–O bond [370]. Hence, optimization of the Si:Al ratio is highly essential to stabilize the polycrystalline structure, along with to enhance the wettability and membrane surface charge, resulting in high flux and salt rejection. In case of Al-rich zeolite membranes, functionalization of the zeolite cage can also be carried out to enhance the membrane performance. Instead of the extensive utilization of hydrophilic membranes imparting higher water fluxes, the hydrophobic character of the zeolite sub-nanometer pore structure plays the pivotal role to offer a desired internal surface chemistry, facilitating the higher mass flow across the zeolite membranes [347]. Duke et al. [334] reported seawater desalination using MFI zeolite membranes of variable Si:Al ratios.

The defect-free zeolites are highly preferable as the diffusion can only take place through the intra-crystalline pores within the zeolite cage resulting in the excellent separation performance. Unfortunately, the preparation of such defect-free zeolite thin film with no micro-defect is always hampered by the defects of synthesis including the insufficient intergrowth of crystals. Moreover, since zeolite membranes are polycrystalline films of nano-sized inter-crystal pores, the interactions between zeolite and ions in feed solution restrict the permeance of some ions into the zeolitic pores during the passage through pores and boundaries reducing the permeability. Additionally, during the attachment of ions onto the external surface and microporous inter-crystal boundaries, coinciding double layers tend to accumulate that severely hampered the ion transportation [329]. In the maximum situations, the ion rejection and water flux are related to the size and charge density of the solute ion, double layer thickness, ionic strength, and temperature of the solution. In this context, Zhou et al. reported that FAU zeolites with greater hydrophilicity can overcome the water flux and ion selectivity tradeoff via producing improved water adsorption and diffusion through their relatively large pore size [349].

#### 5.1.4. Silica Membranes

Another widely used inorganic membrane for salt desalination is the silicate membrane. Li et al. [329] reported a silicalite membrane of ~1 nm pore diameter and high chemical stability for desalination. For a 0.1 (M) NaCl solution, the said silicalite membrane showed time-dependent transient water flux and Na^+^ rejection up to 0.112 kg m^−2^ h^−1^ and 76.7%, respectively. For a mixture of salt solution containing 0.1 (M) each of NaCl, KCl, NH_4_Cl, CaCl_2_, and MgCl_2_, the water flux of 0.058 kg m^−2^ h^−1^ and rejections for Na^+^, K^+^, NH_4_^+^, Ca^2+^, and Mg^2+^ up to 58.1, 62.6, 79.9, 80.7, and 88.4%, respectively, were observed. Li et al. also investigated the influence of salt concentration, especially counter cations, on water flux and ion rejection [371]. A drastic decline of Na^+^ rejection and water flux, in the aid of multivalent cations, such as Ca^2+^ and Al^3+^, was observed because of the adsorption of multivalent cations. Due to the screening effect of the adsorbed cations on the surface charge, an increment of the effective pore diameter was observed. Fortunately, such increment in pore diameter enhanced the permeation of small Na^+^ ions, resulting in the increase in Na^+^ rejection. Skluzacek et al. [372] reported the salt rejection using an iron-modified silica membrane of 15 Å pore diameter. Interestingly, salts rejections within 60–80% were reported for NaCl and Na_2_SO_4_ solutions, whereas up to 10% salt rejection was observed for solutions of MgCl_2_ and MgSO_4_. Poor salt rejection in Mg^2+^-salt solutions could be explained on the basis of the charge exclusion mechanism induced by charge reversal because of the selective adsorption of Mg^2+^. Alami-Younssi et al. [373] reported metal-complex desalination using a γ-alumina membrane with a 7 Å pore diameter, in which fluxes and salt rejections were found to be the functions of pH of solution as well as size charge of the complexes. Fluxes were influenced by the electrostatic interactions between complex and membrane surface, whereas the rejections depend on size and charge of the complex. Samuel de Lint et al. [374] reported the electrolyte separation from 1 (M) NaCl using γ-alumina-supported silica membrane bearing 8 Å pore diameter. The Na^+^ and Cl^−^ rejections based on charge exclusion mechanism strongly depend on the pH of the solution. Gazagnes et al. [375] reported seawater desalination using a zirconia membrane with a 50 nm pore diameter, showing high rejection and flux of >95% and 38.4 L day^−1^ m^−2^.

Duke et al. [334] reported PV desalination using α-alumina, γ-alumina, and silica membranes of 5000, 40, and 3–5 Å pore diameters, respectively, in which water flux and NaCl rejection were functions of salt concentration and PV time. However, water flux and NaCl rejection were found to depend closely on membrane pore size and surface charge. The silica membranes comprising of the pore diameters within 3–5 Å and the pH at point of zero charge (i.e., pH_PZC_) of 2 showed low fluxes and positive rejections, whereas γ-alumina membrane carrying 40 Å pore diameter and a similar pH_PZC_ manifested much higher fluxes and negative rejections. Duke et al. achieved more than 97% rejection [334] for seawater desalination using MFI zeolite membranes of varying Si:Al ratios. The variation in flux was explained by joint pore structure/surface charge/ion exchange/thermal expansion mechanism and the rejection through joint charge exclusion/size exclusion/surface evaporation mechanism. In another study, the same group reported high NaCl rejection of ~97% and water flux of 3 kg m^−2^ h^−1^ in PV using a carbonized template silica membrane [109]. Malekpour et al. [111] reported very high salt rejections up to 99% during desalination of radioactive solutions (i.e., 0.001 (M) of Cs^+^, Sr^2+^, and MoO_4_^2−^) using NaA zeolite membranes. With the increase in PV time, the total flux was found to decrease because of the precipitation of salts into the non-zeolitic pores. Again, Khajavi et al. investigated the desalination of seawater using hydroxyl-sodalite membrane comprising of small diameter = 2.65 Å. The membrane showed high rejections in seawater and NaCl and NaNO_3_ solutions. The water flux for seawater was observed to be higher than that for pure water because of the exchange of O^−^ with Cl^−^. In this context, Khajavi et al. [376] observed the gradual decrease in water flux with the increase in NaOH concentration for the hydroxyl-sodalite membrane.

## 6. Conclusions and Future Perspectives

PV technology employed for the separation and purification of water and organic components secures a unique position in food, chemical, and pharmaceutical industries. Despite several limitations, hundreds of PV companies have been successfully involved in chemical separation throughout the world. Recent development in PV methodology has visualized a clean and invulnerable opportunity that propels the awareness of energy conservation and environmental protection, accompanied by the maximum recovery from the waste stream and minimum waste generation worldwide. The process has been employed successfully to breakdown the azeotropic mixtures and separation of close boiling solvents, recovery of trace elements or contaminants from the liquid mixtures, along with handling heat sensitive bio-components, and found to be the most energy-efficient and clean technology. In spite of the significant advancement in many countries during the last few decades, rapid industrial and economic growth, shortage in energy resources, intensifying necessities of increasing populations are triggering enormous danger to the surrounding environment. Indeed, substantial research going on in the field of membrane based desalination emphasizing use of the minimum energy, along with the excellent efficiency during generation of pure water.

Although conventional distillation methods have desalinated water for a long time, the low energy consumption, high selectivity, lower capital and maintenance costs, and compact modular design make the membrane-based desalination technologies more attractive and the preferred choice instead of thermal desalination techniques. The RO process has already been commercialized throughout the world for desalination purposes. However, manufacturing robust, but thinner, chemically stable, and fouling resistant RO composite membranes remains the challenging task. Moreover, the RO process required significant amount of energy to overcome the osmotic pressure, which increases largely with water salinity. In contrast, PV has emerged as a high potential alternative method for energy efficient desalination. The energy conversation in PV desalination, along with its ability to handle high salinity water, was found to be very high. The difference in solubility and diffusivity of feed components allows a particular component to pass through the PV membrane. Similar to MD, permeate can be removed from the downstream side by applying vacuum or sweep gas. It is noteworthy to mention that the PV process exhibits low permeation rate, and is usually applied to remove the component, which is present in smaller amount in the feed mixture. However, in the case of water desalination, the membranes in PV are usually hydrophilic and preferentially allow water to absorb into the membrane matrix through specific dipole-dipole interactions or hydrogen bonding, followed by diffusion across the membrane. In PV, preferential membrane affinity, membrane morphology, and thickness play important roles for the permeation of water. The lower water permeation rate through the membrane matrix is attributed to the high mass transfer resistance appeared during the diffusion step, a critical issue hindering the commercialization of the PV process. Moreover, the ‘trade-off’ relationship between flux and selectivity limits the use of membranes at the industrial scale.

Although commercial applications of PV have been employed successfully in solvent dehydration, the commercial application of PV technology in desalination has not been found to date. The availability of superior commercial membranes is the most vital restraint in the development and employment of large-scale PV desalination. However, with the development of high-affinity PV membranes, along with suitable morphology for water transport, containing ideal FFV space, the water permeation rate could enhance over the theoretical values calculated from transport equations. Incorporation of advanced nanostructured materials not only provide mechanical strength, but also exhibited superior performances in terms of water flux and salt rejection. The employment of these nanomaterial-enabled membranes will continue to capture the growth of cost efficient PV system and stable modular design to reduce the capital and operational cost, help to construct a robust desalination technology with the minimum energy requirement and environmental impact. Moreover, the application of PV is less extensive, for which salt rejection rate, especially for monovalent ions, are observed to be very high, usually above 99%, wherein the performances do not depend much on the operating conditions. Additionally, being a water selective membrane process, PV does not allow any other organic VOCs or impurities present in the feed system. The major problems associated with other membrane-based separation systems, such as membrane wetting, concentration polarization, temperature polarization, membrane fouling, and pore-plugging, are not severe in PV, helping the attainment of consistent membrane performances for a longer period of time. PV could be feasible in treating highly concentrated salt water, including produced water from mineral oil and natural gas plants, as the energy requirement of PV dehydration does not depend on the salt concentration of feed. The water recovery can possibly be very high so that the highly concentrated PV-reject may be utilized concomitantly resulting in zero liquid discharge and the minimum environmental pollution.

So far, the researchers have made significant improvement in PV desalination process and several difficulties need to be addressed before the attainment of successful implementation. The development of proper membrane and modular design, optimization of operating conditions, increase in mass transfer coefficient could possibly enhance the competitiveness of the process, which eventually led to finding a solution to global water scarcity. For further attainment of a high-performance PV-desalination system showing elevated salt-rejection efficiency, attempts can be made to develop long-lasting, multi-stage, multi-functional, fouling resistant, stimuli-responsive, smart nanocomposite hybrid membranes capable of long-term, high-flux pervaporative desalination of multi-component concentrated salty-water.

## Figures and Tables

**Table 1:** Chronological order relating to the development of pervaporative desalination by different membranes.

Year	Polymer/Composite/ NCP Membranes	Fabrication Process	Advantages	Drawbacks	Ref
1996	Sulfonated polyethylene hollow fibers	Not reported	a. Superior control of operational parameters; b. Optimized pressure drop and reduced energy for water/ air circulation c. Desalinated water is free of organic substances	a. low production rates per unit area	[41]
2005	polyetheramide-based polymer film	Yet to be disclosed	a. Utilization of renewable, non-conventional energy resources b. Simple construction, operation, and maintenance c. Utilization of dense membranes prevent wetting induced salt leakage and pore-plugging problems are anticipated not to occur	a. yet to be commercialized b. low production rates per unit area	[43]
2008	NaA zeolite	Direct hydrothermal synthesis on porous α-alumina support	a. Higher thermal, chemical and mechanical resistance b. Improved SR ^ab^ efficiency	higher production cost	[108]
2009	CTS ^a^ membranes	Two-step sol-gel catalyzed process	Maximum NaCl rejection and flux are 97% and 3 kg m^−2^ h^−1^, respectively for membrane derived from the longest carbon chain (C16) surfactant	a. unstable during desalination, as water interacted with the silanol groups and enlarged pore sizes of microporous silica film.	[109]
2010	Hydroxy sodalite membrane	Surface of a polished-alumina disk (25 mm diameter, 2 mm thickness, 80 nm top layer pore size, and 150 nm bottom layer pore size)	–	a. poor particle dispersion driven inferior polymer-inorganic interactions and structural defects in the membrane	[79]
2011	NaA zeolite	Secondary growth process, with a single-channel porous α-alumina tube applied as a support.	a. Improved thermal, chemical, and mechanical resistance b. Appreciable SR efficiency	a. higher production cost b. poor particle dispersion driven inferior polymer-inorganic interactions and structural defects in the membrane	[81]
2011	Hybrid PVA ^b^/MA ^c^/silica membrane	Aqueous sol-gel route	a. Crosslinking among three components resulting higher crosslinking density and better SR	–	[46]
2011	Silicalite-polyamide composite membranes	Interfacial polymerization	a. Capable to desalinate saline water of exceptionally high initial concentration at a significantly higher flux and SR	–	[110]
2011	LTA^d^ and MFI zeolite	Hydrothermal synthesis on the surface of an α-alumina porous support.	a. Better thermal, chemical, mechanical resistances b. Improved SR efficiency	–	[111]
2011	Templated silica	Interfacial polymerization on a commercial polysulfone substrate	–	–	[112]
2012	hydrophilic polyester tubular pervaporative membrane	grown hydrothermally on the surface of an α-alumina porous support	–	–	[102]
2012	S-1 ^e^ and ZSM-5 ^f^ membranes	Secondary growth on tubular ceramic supports	a. Mechanically stronger and durable	a. higher production cost	[55]
2012	Dense natural zeolite	Membranes were sliced as sheets from the as-mined material after a visual inspection	a. High temperature stability b. Higher thermal, chemical, mechanical resistances, along with significant SR efficiency	a. poor particle dispersion driven inferior polymer-inorganic interactions and structural defects in the membrane	[113]
2014	Cellulose triacetate membrane	Dip-coating membrane	–	–	[101]
2014	Natural zeolite clinoptilolite-phosphate composite	Dry powder pressing followed by high temperature steaming	a. Higher thermal, chemical, mechanical resistances, along with significant SR efficiency	–	[107]
2014	PVA ^b^(uncrosslinked)/PAN ^g^/PET ^h^	Electrospraying and electrospinning	a. PAN nanofiber provided necessary mechanical strength	–	[80]
2015	MA ^c^ crosslinked PVA ^b^/PVSF ^j^ hollow fiber	Direct spinning and phase inversion	a. PVSF hollow fiber provided mechanical strength, chemical resistance, and thermal stability	–	[114]
2015	Mesostructured CTAB ^k^-silica membrane	–	–	–	[115]
2015	GO ^l^/PAN ^g^ composite membrane	Vacuum filtration-assisted assembly method	a. Exfoliated distribution of GO particles	a. higher production cost b. poor particle dispersion driven inferior polymer-inorganic interactions and structural defects in the membrane	[50]
2016	GOF ^m^ membrane	Vacuum filtration of GOF suspension	a. Outstanding water permeability b. Preferential water adsorption ability and fast water diffusivity	a. higher production cost b. poor mechanical strength and susceptible to destruction during practical applications.	[116]
2016	GOF ^m^ membranes PDI ^n^-modified α-Al_2_O_3_	Vacuum filtration of GOF suspension	a. Thick GO membranes equipped with enhanced mechanical stability.	–	[116]
2017	nanohybrid GO ^l^/PI ^o^ MMMs ^p^	Phase inversion in a water coagulation bath	a. higher desalination performance b. stable under harsh conditions	a. low packing density. b. higher production cost	[117]
2017	PVA ^b^-SiO_2_/PVSF ^j^ hollow fiber	Direct spinning and phase inversion	a. PVSF hollow fiber provided mechanical strength, chemical resistance, and thermal stability b. SiO_2_ filler mediated crosslinks	a. complicated and time-consuming to fabricate	[118]
2017	PEBA ^q^/PAN ^g^/PE ^r^, PEBA ^q^/PSF ^s^/PE ^r^, PEBA ^q^ + NaX/PSF ^s^/PE ^r^	Solution mixing and casting	a. Soft and flexible segments b. High sorption of water vapor	a. yet to be scaled up and commercialized	[119]
2017	zeolite 3A/PEBA ^q^	Solution mixing and casting	a. Molecular sieving effect of the zeolite 3A cages improved SR b. Better chemical resistance and higher SR efficiency	a. higher production cost	[120]
2017	GNPs ^t^/PEBA ^q^	Solution mixing and casting	a. superior antifouling property	–	[121]
2017	SSA ^u^ crosslinked PVA ^b^/PAN ^g^	Solution mixing and casting	a. Improved flux owing to Sulfonic acid bearing crosslinks b. PAN provided mechanical strength and thermal stability	water-soluble PVA is to be crosslinked to increase the stability in water	[122]
2017	GA ^i^ crosslinked PVA ^b^/PVDF ^v^	Dip coating and cast-coating.	a. Excellent storage stability, anti-fouling properties, and cleaning efficiency	a. low desalination productivity and poor operational stability with brine feeds.	[123]
2018	thin PIM-1 ^w^ membrane	Dispersing GO into PI through wet phase inversion method	a. PI possesses good thermal and mechanical stability, easy processing and good solubility in various common solvents	a. PI membrane was affected by polymer concentration, evaporation time and post casting annealing, substantiated by the molecular weight cut-off curve	[124]
2018	MWCNT-PVA ^x^	Solution mixing and casting	Good film formability; higher hydrophilicity introduced by OH and COOH; superior antifouling property; improved SR, durability, electrical conductivity; higher adsorption and water fluxes	costly	[125]
2018	GO ^l^/PI ^o^ hollow fiber	Direct spinning and phase inversion	–	–	[126]
2018	GO ^l^/CS ^y^	Solution mixing and casting	a. mechanically stable via covalent crosslinking between epoxy of GO with amine of CS	–	[114]
2018	PMDA ^z^ crosslinked PVA ^b^/PAN ^g^	Solution mixing and casting	a. PAN provided mechanical strength	–	[127]
2018	SPTA ^aa^ crosslinked PVA ^b^/PAN ^g^	Solution mixing and casting	a. Sulfonic acid containing crosslinks improved flux b. PAN provided mechanical strength	–	[128]

^a^ carbon template silica, ^b^ polyvinyl alcohol, ^c^ maleic acid, ^d^ linde type A, ^e^ silicalite-1, ^f^ Zeolite Socony Mobil–5, ^g^ polyacrylonitrile, ^h^ polyethylene terephthalate, ^i^ glutaraldehyde, ^j^ polyvinylsulfone, ^k^ cetyltrimethyl ammonium bromide, ^l^ graphene oxide, ^m^ graphene oxide framework, ^n^ 1,4-phenylene diisocyanate, ^o^ polyimide, ^p^ mixed matrix membranes, ^q^ polyether block amide, ^r^ polyester, ^s^ polysulfone, ^t^ graphene nanoplates, ^u^ sulfosuccinic acid, ^v^ polyvinylidene difluoride, ^w^ polymers of intrinsic microporosity, ^x^ multi-walled carbon nanotubes loaded polyvinyl alcohol membranes, ^y^ chitosan, ^z^ pyromellitic dianhydride, ^aa^ 4-sulfophthalic acid, and ^ab^ salt rejection.

**Table 2:** Properties of polymeric membranes and their performances in pervaporative desalination.

Composite/NCP Membranes	Components	T_g_	Thickness (μm)	Degree of Swelling (%)	Contact Angle	Ref
PEBA ^a^/PAN ^b^/PE ^c^	PEBA ^a^, PAN ^b^, PE ^c^	–	11.0	21.8	53.0	[119]
PEBA ^a^/PSF ^d^/PE ^c^	PEBA ^a^, PSF ^d^, PE ^c^	–	–	21.8	53.0	[119]
PEBA ^a^ + NaX ^e^/PSF ^d^/PE ^c^	PEBA ^a^ + NaX ^e^, PSF ^d^, PE ^c^	–	–	21.2	53.0	[119]
PVA noncrosslinked TFNPVC ^f^	PVA ^g^, PAN ^b^, PET ^h^	–	0.6	180.5	48.2	[80]
PVA crosslinked TFNPVC ^f^	PVA ^g^, PAN ^b^, PET ^h^	–	0.7	14.5	63.5	[80]
S-PVA ^i^ (1:10)/PAN films	PAN ^b^, PVA ^g^, SPTA ^j^	–	0.8	150.0	46.4 ± 3.4	[128]
cellulose triacetate	cellulose triacetate	–	10.0	–	46.1 ± 3.0	[101]
cellulose acetate	cellulose acetate	–	20.0–25.0	99.7	–	[130]
PE ^c^	PE ^c^	–	750.0	60.0	–	[102]
PVA ^g^/MA ^k^ (M-1)	PVA ^g^, MA ^k^	366.0–401.0	0.1	–	71.5	[114]
PVA ^g^/MA ^k^ (M-2)	PVA ^g^, MA ^k^	366.0–401.0	0.1	–	65.1	[114]
PVA ^g^/MA ^k^ (M-3)	PVA ^g^, MA ^k^	366.0–401.0	0.3	–	63.2	[114]
PVA ^g^/MA ^k^ (M-4)	PVA ^g^, MA ^k^	366.0–401.0	0.4	–	61.1	[114]
PVA ^g^/MA ^k^ (M-5)	PVA ^g^, MA ^k^	366.0–401.0	0.9	–	56.5	[114]
PVA ^g^/MA ^k^ (M-6)	PVA ^g^, MA ^k^	366.0–401.0	1.0	–	52.7	[114]
clinoptilolite-phosphate	MKP ^l^, MgO	–	1300.0	–	–	[107]
CTAB ^m^-silica	CTAB ^m^, SiO_2_	–	0.2	–	–	[115]
polyether amide	polyether amide	–	40.0	–	–	[43]
NaA zeolite membrane	NaA zeolite membrane	–	–	–	–	[81]
GO ^n^/PAN ^b^	GO ^n^, PAN ^b^	–	0.1–1.4	–	–	[50]
2D MXene	M_n+1_X_n_T_x_, where n=1, 2, or 3, M=early transition metal, X=C/N, and T=surface group (OH, O, or F)	–	0.1	–	45.9	[133]
GO ^n^/PVA ^b^/PVDF	0.2 wt% GO incorporated PVA/PVDM composite	60.0	100.0	38.2 ± 2.1	37.1 ± 2.3	[134]
LiCl-SPVA	LiCl, SPTA, PVA	–	33.1	–	–	[135]
PEBA ^o^	PEBA ^o^	–	100.0	–	86.0	[120]
Zeolite 3A incorporated PEBA composite membrane	Zeolite 3A, PEBA ^o^	–	100.0	–	63.0–71.0	[120]
CS ^o^ membrane	CS	140.6	10.0–13.0	–	85.0	[136]
CS/GO MMM ^p^	CS, GO	143.5–145.3	10.0–13.0	–	77.5	[136]
PVA ^g^ dense film	PMDA ^q^, PVA ^g^	–	2.0	–	42.1	[127]
PVA ^g^/PAN ^b^ composite PV membrane (M-a)	0% of the mole concentrations of –COOH group of the hydrolyzed PMDA ^q^, PVA ^g^, PAN ^b^	75.0	2.0	–	–	[127]
PVA ^g^/PAN ^b^ composite PV membrane (M-b)	5% of the mole concentrations of –COOH group of the hydrolyzed PMDA ^q^, PVA ^g^, PAN ^b^	75.0	2.0	170.5	42.1	[127]
PVA ^g^/PAN ^b^ composite PV membrane (M-c)	10% of the mole concentrations of –COOH group of the hydrolyzed PMDA ^q^, PVA ^g^, PAN ^b^	75.0	2.0	110.9	57.4	[127]
PVA ^g^/PAN ^b^ composite PV membrane (M-d)	20% of the mole concentrations of –COOH group of the hydrolyzed PMDA ^q^, PVA ^g^, PAN ^b^	–	2.0	88.0	55.0	[127]
PVA ^g^/PAN ^b^ composite PV membrane (M-e)	30% of the mole concentrations of –COOH group of the hydrolyzed PMDA ^q^, PVA ^g^, PAN ^b^	–	2.0	90.0	52.5	[127]
PVA ^g^/MA ^k^/ Silica	PVA ^g^, MA ^k^, Silica	–	10.0	36.0 ± 5.0	51.5	[100]
Polyether ester	Polyether ester	–	160.0	–	–	[137]
FAS ^r^-Al_2_O_3_^s^	1H,1H,2H,2H-perfluorodecyltriethoxysilane, alumina	–	23.0	–	–	[138]
FAS-TiO_2_^t^	1H,1H,2H,2H-perfluorodecyltriethoxysilane, titania	–	23.0	–	–	[138]
CC ^u^ membrane	Cotton	–	30.0	44.0	–	[131]
WC ^v^ membrane	Wood	–	30.0	28.0	–	[131]
CDA ^w^ membrane	Cellulose diacetate	–	3.0–5.0	6.5	–	[131]
GFBC-10^x^	Bacterial cellulose	–	240.0	–	–	[131]
BC-D ^y^	Bacterial cellulose	–	40.0	–	–	[131]
Sulfonated PE ^z^	Sulfonated polyethylene	–	100.0	25.0–36.0	–	[41]
Quaternized PE ^z^	Quaternized polyethylene	–	70.0	31.5	–	[42]
Quaternized PE ^z^	Quaternized polyethylene	–	170.0	36.5	–	[42]
S-PVA/PAN ^b^	SSA ^aa^, PVA ^a^, PAN ^b^	113.0	4.9	83.5 ± 5.1	77.1 ± 3.0	[122]
PVA ^g^	PVA ^g^	84.0	4.9	194.3 ± 10.7	44.3 ± 2.7	[122]
PVA ^g^/MA^k^	PVA ^g^, 20% MA ^k^	94.0	5.0	61.0 ± 5.0	59.4 ± 2	[139]
PVA ^g^/20MA ^k^/ 10Silica	PVA ^g^, 20% MA ^k^, 10% Silica	103.0	5.0	22.0 ± 2.0	63.5 ± 2	[139]
PVA ^g^/20MA ^k^/ 25Silica	PVA ^g^, 20% MA ^k^, 25% Silica	107.0	5.0	11.0 ± 1.0	79.4 ± 2	[139]
PEBA ^a^		–	0.1	–	86.0	[121]
2GNPs ^ab^/PEBA ^a^	2.0 wt% GNP, PEBA ^a^	–	0.1/0.2	–	80.0	[121]
5GNPs ^ab^/PEBA ^a^	5.0 wt% GNP, PEBA ^a^	–	0.2	–	75.0	[121]
0.3MWCNT/PVA ^ac^	0.3 wt% MWCNT ^ac^, PVA ^g^	–	100.0	275.0–350.0		[100]
PI ^ad^ hollow fiber	14.6 wt% PI ^ad^, 2.0 wt% PVP ^ae^, 9.1 wt% EtOH, 74.3 wt% NMP ^af^	–	1000.0	–	92.0	[126]
GO ^n^/PI ^ad^ hollow fiber	1.0 wt% GO, 14.4 wt% PI ^ad^, 2.0 wt% PVP ^af^, 9.0 wt% EtOH, 73.6 wt% NMP ^af^	–	1000.0	–	59.0	[126]
CS ^o^	0.1 wt% GO ^n^, CS ^o^	140.6	–	–	85.0	[136]
1GO ^n^/CS ^o^	1.0 wt% GO ^n^, CS ^o^	143.5	–	–	77.5	[136]
2GO ^n^/CS ^o^	2.0 wt% GO ^n^, CS ^o^	145.3	–	–	77.0	[136]
PVA ^g^-SiO_2_/ PVSF ^ag^ hollow fiber	52.4:1:1:(1000-67) = C_2_H_5_OH: TEOS: PVA^g^:H_2_O (*w*/*w*)	396.0	0.2–1.1	–	–	[118]

^a^ poly(ether block amide), ^b^ polyacrylonitrile, ^c^ polyester, ^d^ polysulfone, ^e^ sodium halide, ^f^ three-layer thin film nanofibrous PV composite, ^g^ polyvinyl alcohol, ^h^ polyethylene terephthalate, ^i^ sulphonic acid functionalized polyvinyl alcohol, ^j^ 4-sulfophthalic acid, ^k^ maleic acid, ^l^ monopotassium phosphate, ^m^ cetyltrimethylammonium bromide-silica, ^n^ graphene oxide, ^o^ chitosan, ^p^ chitosan/graphene oxide mixed matrix membrane, ^q^ pyromellitic dianhydride, ^r^ fluoroalkylsilane-ceramic, ^s^ alumina grafted fluoroalkylsilanes-modified PV ceramic nanofiltration membranes, ^t^ titania grafted fluoroalkylsilanes-modified PV ceramic nanofiltration membranes, ^u^ cotton cellulose, ^v^ wood cellulose, ^w^ cellulose diacetate, ^x^ bacterial cellulose containing 10% residual water, ^y^ bacterial cellulose dried to constant weight, ^z^ sulfonated polyethylene hollow fibers, ^aa^ sulfosuccinic acid, ^ab^ graphene nanoplates, ^ac^ multi-walled carbon nanotubes loaded polyvinyl alcohol membranes, ^ad^ polyimide, ^ae^ polyvinylpyrrolidone, ^af^ N-methyl-2-pyrrolidinone, ^ag^ polyvinylsulfone, and ^ah^ tetraethylorthosilicate.

**Table 3:** Performances of polymeric membranes in pervaporative desalination.

Composite/NCP Membranes	Thickness (μm)	Feed Temperature (°C)	Feed Flow Rate (mL min^−1^)	Feed Concentration (ppm)	Activation Energy (kJ mol^−1^)	Permeate Pressure	Flux (kg m^−2^h^−1^)	Salt Rejection (%)	Ref
PVA ^a^/20MA ^b^/10Silica	5	22	–	–	–	0.80	5.51	>95.50	[139]
PVA ^a^/20MA ^b^/25Silica	5	22	–	–	–	0.80	>3.65	>95.50	[139]
PVA ^a^/5MA ^b^/10Silica	20 ± 1	22–65	30	2000–50,000 NaCl	23.80–20.10	0.80	2.50–11.70	99.90 (max)	[100]
GO ^c^/PAN ^d^	–	30	–	2000/35,000/50,000/100,000	22.19	–	16.84/14.31/13.56/ 11.23	>99.80	[50]
GO ^c^/PAN ^d^	0.003–1.4	90	–	35,000	22.19	–	65.10	>99.80	[50]
PEBA ^e^	120 ± 5	65	–	–	–	0.50	–	99.12	[121]
3GNPs ^f^/PEBA ^e^	72/120 ± 5/181	65	–	–	–	0.50	5.12/3.61/2.73	>99.89	[121]
3GNPs ^f^/PEBA ^e^	120 ± 5	35	–	–	–	0.50	2.58	99.94	[121]
MWCNT-PVA ^g^	100	40	–	NaCl (1000/1500/2000) + MgCl_2_ (200) + KCl (200)	–	–	–	>92.00	[125]
GO ^c^/PI ^h^ hollow fiber	1000	45	–	35,000 sea water [Na^+^ (2067) K^+^ (323) Mg^2+^(872) Ca^2+^(247), F^−^ (188), Cl^−^ (3132), PO_3_^−^(1025)]	18.76	–	6.40	>99.80 [Na^+^ (99.90) K^+^ (99.80) Mg^2+^(99.90) Ca^2+^(99.90), F^−^ (99.80), Cl^−^ (99.90), PO_3_^−^ (99.80)]	[126]
GO ^c^/PI ^h^ hollow fiber	1000	60	–	Do	Do	–	8.10	Do	[126]
GO ^c^/PI ^h^ hollow fiber	1000	75	–	20,000/35,000/100,000	Do	–	17.50/11.50/4.20	Do	[126]
GO ^c^/PI ^h^ hollow fiber	1000	90	–	35,000 sea water [Na^+^ (2067) K^+^ (323) Mg^2+^(872) Ca^2+^(247), F^−^ (188), Cl^−^ (3132), PO_3_^−^(1025)]	Do	–	15.60	Do	[126]
PI ^h^	1000	75	–	Do	–	–	1.90	–	[126]
PEBA ^e^/PAN ^d^/PE ^i^	–	40/50/60	–	760 NaCl	–	4.00	>3.00/4.93/7.63	>93.00	[119]
PEBA ^e^/PSF ^j^/PE ^i^	–	50	–	Do	–	Do	1.24	>93.00	[119]
PEBA ^e^+NaX/PSF ^j^/PE ^i^	–	50	–	Do	–	Do	1.30	>93.00	[119]
PVA ^a^(GA ^k^ crosslinked)/PAN ^d^/PET ^l^	–	25	–	5000/35,000/50,000	–	0.10	8.53/7.36/5.81	99.90/99.80/99.80	[80]
SPTA ^m^ crosslinked PVA ^a^/PAN ^d^	0.8 (thickness of SPTA ^m^ crosslinked PVA ^a^)	70	–	35,000 NaCl	–	0.10	49.30 ± 1.10	99.80	[128]
SPTA ^m^ crosslinked PVA ^a^/PAN ^d^	0.8/1.06/1.84/2.56/4.48/4.86/17.2/33/46.3/76 (thickness of SPTA ^m^ crosslinked PVA ^a^)	30	–	35,000 NaCl	–	0.10	14.11/11.23/10.08/9.50/9.42/8.64/8.35/6.76/5.18/3.74	99.80 ± 0.10	[128]
SPTA ^m^ crosslinked PVA ^a^/PAN ^d^	0.8	30	–	100,000 NaCl	–	0.10	7.80 ± 0.30		[128]
20zeolite 3A/PEBA ^e^	100	40	–	10,000/30,000/100,000 NaCl	–	–	3.30/3.10/1.98	99.63	[120]
20zeolite 3A/PEBA ^e^	100	60	–	30,000 NaCl	–	–	4.33	99.16	[120]
PEBA ^e^	100	40	–	30,000 NaCl	–	–	2.07	>99.50	[120]
GO ^c^/CS ^n^	–	81	1000	50,000/100,000 NaCl	31.28 ± 1.09/ 32.95 ± 2.37	0.80	30.00/27.60	99.99	[136]
GO ^c^/CS ^n^	–	75	1000	50,000 NaCl	Do	6.00	25.80	99.99	[136]
GO ^c^/CS ^n^	–	60	1000	35,000/100,000 NaCl	Do	6.00	17.70/16.20	99.99	[136]
CS ^n^	–	81	1000	50,000/ 100,000 NaCl	37.94 ± 1.19/ 42.90 ± 1.93	6.00	–	–	[136]
SSA ^o^ crosslinked PVA ^a^/PAN ^d^	4.9 (SSA ^p^ crosslinked PVA ^a^ layer)	70	–	35,000	–	0.10	27.90	99.80	[122]
SSA ^o^ crosslinked PVA ^a^/PAN ^d^	4.9/18.4 (SSA ^p^ crosslinked PVA ^a^ layer)	30	–	35,000	–	0.10	7.90/6.40	99.80	[122]
SSA ^o^ crosslinked PVA ^a^/PAN ^d^	4.9 (SSA ^p^ crosslinked PVA ^a^ layer)	30	–	100,000	–	0.10	4.50	–	[122]
20PMDA ^p^ crosslinked PVA ^a^/PAN ^d^	2 (PMDA ^p^ crosslinked PVA ^a^ layer)	70	–	35,000	23.60	0.10	32.26	99.98	[127]
5PMDA ^p^ crosslinked PVA ^a^/PAN ^d^	2 (do)	50	–	35,000	–	0.10	9.88	99.96	[127]
10PMDA ^p^ crosslinked PVA ^a^/PAN ^d^	2 (do)	50	–	35,000	–	0.10	12.32	99.98	[127]
20PMDA ^p^ crosslinked PVA ^a^/PAN ^d^	2 (do)	50	–	35,000	–	0.10	16.47	99.98	[127]
30PMDA ^p^ crosslinked PVA ^a^/PAN ^d^	2 (do)	50	–	35,000	–	0.10	16.02	99.98	[127]
PVA ^a^-SiO_2_/PVSF ^q^ hollow fiber	0.22 ± 0.03	60	–	2000/30,000/50,000	–/14.44/14.74	–	20.60 ± 0.45/10.40 ± 0.22/8.80 ±0.20	99.90	[118]
PVA ^a^-SiO_2_/PVSF ^q^ hollow fiber	0.30 ± 0.03	60	–	Do	–/15.05/16.47	–	19.80 ± 0.40/ 9.70 ± 0.24/7.90 ± 0.18	99.90	[118]
PVA ^a^-SiO_2_/PVSF ^q^ hollow fiber	0.60 ± 0.05	60	–	Do	–/16.63/20.05	–	17.20 ± 0.40/8.10 ± 0.22/6.70 ± 0.17	99.90	[118]
PVA ^a^-SiO_2_/PVSF^q^ hollow fiber	0.85 ± 0.07	60	–	Do	–/18.52/23.49	–	15.30 ± 0.33/7.00 ± 0.23/5.90 ± 0.18	99.90	[118]
PVA ^a^-SiO_2_/PVSF ^q^ hollow fiber	1.13 ± 0.11	60	–	Do	–/22.01/24.48	–	11.90 ± 0.29/5.80 ± 0.25/4.70 ± 0.26	99.90	[118]
MA ^b^ crosslinked PVA ^a^/PVSF ^q^ hollow fiber	0.10	71	–	30,000/40,000/50,000	25.80/25.40/25.39	–	4.60–7.40/–/–	99.90	[114]
MA ^b^ crosslinked PVA ^a^/PVSF ^q^ hollow fiber	0.14	71	–	Do	25.80/25.06/25.06	–	Do	99.90	[114]
MA ^b^ crosslinked PVA ^a^/PVSF ^q^ hollow fiber	0.27	71	–	Do	24.51/24.60/24.41	–	Do	99.90	[114]
MA ^b^ crosslinked PVA ^a^/PVSF ^q^ hollow fiber	0.40	71	–	Do	23.72/24.38/24.06	–	Do	99.90	[114]
MA ^b^ crosslinked PVA ^a^/PVSF ^q^ hollow fiber	0.85	71	–	Do	23.80/23.62/23.51	–	Do	99.90	[114]
MA ^b^ crosslinked PVA ^a^/PVSF ^q^ hollow fiber	1.03	71	–	Do	23.46/23.10/23.07	–	Do	99.90	[114]
nonporous films of hydrated cellulose	–	–	–	–	–	0.02	0.91–1.90	100.00	[142]
CA ^r^ powder based membranes	20–25	50–80	–	40,000, 48,000, 50,000, and 140,000 NaCl	21.71, 19.83, 15.94, and 20.21	–	5.97–10.00	99.70	[130]
cellulose triacetate	10	50	–	100,000	–	air sweep	2.30	99.00	[101]
quaternized PE ^i^, anion exchanger	–	45–65	–	0–176,000	–		1.50–3.00		[42]
polyether amide	40	68–70	solar heating	32,000	–	cooler tunnel	0.56	99.99	[43]
deacetylated cellulose acetate	22	70	–	120,000	–	air sweep	4.11	99.90	[211]
cotton cellulose	30	40	–	40,000	–	vacuum; 0.02	4.55–6.70	100.00	[131]
polyester	750	20	–	35,000	–	sand heating	7.10 × 10^−3^	99.84	[102]

^a^ Polyvinyl alcohol, ^b^ maleic acid, ^c^ graphene oxide, ^d^ polyacrylonitrile, ^e^ polyether block amide, ^f^ graphene nanoplates, ^g^ multi-walled carbon nanotubes loaded polyvinyl alcohol membranes, ^h^ polyimide, ^i^ polyester, ^j^ polysulfone, ^k^ glutaraldehyde, ^l^ polyethylene terephthalate, ^m^ 4-sulfophthalic acid, ^n^ chitosan, ^o^ sulfosuccinic acid, ^p^ pyromellitic dianhydride, ^q^ polyvinylsulfone, and ^r^ cellulose acetate.

**Table 4:** Performances of composite/NCP inorganic membranes in pervaporative desalination.

Composite/ NCP Inorganic Membranes	Temperature (°C)	Conditions in Feed Side	Feed Concentration (g L^−1^)	Conditions in Permeate Side	Membrane Thickness (μm)	Flux (kg m^−2^ h^−1^)	Rejection (%)	Ref.
NaA zeolite membrane	69	–	–	–	–	1.90	–	[81]
PVA ^a^/MA ^b^/silica hybrid membrane	22	–	–	–	–	6.93	–	[46]
2D MXene ^c^	65	–	35.00	–	0.06	4.74	99.50	[133]
GO ^d^/PAN ^e^	90	–	100.00	–	0.03–1.40	3.62	99.80	[50]
GO ^d^/PVA ^a^/PVDF ^f^	65	cross-flow velocity = 0.625 ms^−1^	100.00	vacuum pressure ~24,000 Pa	100.00	1.56	99.99	[134]
LiCl-S-PVA ^g^	70	flow rate = 0.2 ms^−1^	35.00	pressure = 100 Pa	33.10	60.80	99.80	[135]
pristine PVA ^a^	20/30/40	atmospheric pressure	35.00	3000 Pa	–	1.49/1.63/1.98	100.00/95.80/95.00	[255]
zeolite 3A-loaded PVA	20/30/40	atmospheric pressure	35.00	3000 Pa	–	1.82/2.36/2.57	100.00/96.10/96.00	[255]
PVA ^a^/PAN ^e^	20	–	5.00	vacuum	0.62	9.04	99.50	[80]
NaA zeolite membrane	69	–	Seawater	vacuum	–	1.90	99.90	[81]
NaA zeolite membrane	77	–	29.00	vacuum	–	4.40	99.90	[81]
LTA ^h^ and MFI zeolite	25	–	0.13	vacuum	15.00	0.20	99.40	[111]
NaA zeolite	20	–	0.10	vacuum	10.00	1.43	99.83	[108]
clinoptilolite-phosphate	95	–	1.40	vacuum	–	15.00	95.00	[107]
silica from TEOS ^i^ and MTES ^j^	25	700 kPa	3.00	vacuum	–	4.70	93.00	[256]
silica from TEOS ^i^ and MTES ^j^	25	700 kPa	3.00	vacuum	–	2.20	99.90	[256]
CTS ^k^	20	–	3.00	vacuum	–	3.20	97.00	[109]
CTAB ^l^-silica	25	–	40.00	vacuum	0.21	2.60	99.90	[115]
templated silica	20	–	35.00	vacuum	0.50	3.70	98.50	[112]
nickel oxide silica	25	–	3.00	vacuum	–	7.00	99.90	[257]

^a^ polyvinyl alcohol, ^b^ maleic acid, ^c^ Ti_3_C_2_T_x_ membranes, ^d^ graphene oxide, ^e^ polyacrylonitrile, ^f^ polyvinylidene difluoride, ^g^ 4-sulfonylphthalic acid cross-linked poly(vinyl alcohol), ^h^ linde type A, ^i^ tetraethyl ortho silicate, ^j^ methyl tri-ethoxy silane, ^k^ carbon template silica, and ^l^ cetyltrimethyl ammonium bromide.

## Abbreviations

TDS	Total dissolved solids
RO	Reverse osmosis
NF	Nanofiltration
UF	Ultrafiltration
MF	Microfiltration
FO	Forward osmosis
PRO	Pressure retarded osmosis
MD	Membrane distillation
PV	Pervaporation
VOCs	Volatile organic compounds
MSFD	Multistage flash distillation
ΔVP	Vapor pressure difference
PVA	Polyvinyl alcohol
MED	Multi effect distillation
TFC	Thin film composite
GO	Graphene oxide
PAN	Polyacrylonitrile
NPs	Nanoparticles
PDMS	Polydimethyl siloxane
CMS	Carbon molecular sieves
MA	Maleic acid
TEOS	Tetraethoxysilane
FFV	Fractional free volume
PET	Polyethylene terephthalate
PMDA	Pyromellitic dianhydride
SSA	Sulfosuccinic Acid
SPTA	4-Sulfophthalic acid
PEBA	Poly(ether block amide)
NCP	Nanocomposite
GNPs	Graphene nanoplates
PI	Polyimide
CS	Chitosan
MMMs	Mixed matrix membranes
PVS	Polyvinyl sulfone
MOFs	Metal organic frameworks
ZIFs	Zeolitic-imidazolate frameworks
PEBAX	Polyether block polyamide
PVDF	Polyvinlyidene fluoride
MWCNT	Multi-walled carbon nanotube
PMMA	Poly(methyl methacrylate)
PE	Polyester
PSF	Polysulfone
NMP	N-Methyl-2-pyrrolidinone
DMF	Dimethylformamide
GA	Glutaraldehyde
T_g_	Glass transition temperature
TS	Tensile strength
EAB	Elongation-at-break
CD	Crosslink density
SR	Swelling ratio
CNTs	Carbon nanotubes
GS	Gas separation
FAS	Fluoroalkylsilanes
CA	Cellulose acetate
LTA	Linde Type A
